# Architecture and Distribution of Introns in Core Genes of Four *Fusarium* Species

**DOI:** 10.1534/g3.117.300344

**Published:** 2017-10-09

**Authors:** Mmatshepho M. Phasha, Brenda D. Wingfield, Martin P. A. Coetzee, Quentin C. Santana, Gerda Fourie, Emma T. Steenkamp

**Affiliations:** * Department of Microbiology and Plant Pathology, Forestry and Agricultural Biotechnology Institute (FABI), Faculty of Natural and Agricultural Sciences, University of Pretoria, 0002 South Africa; † Department of Genetics, Forestry and Agricultural Biotechnology Institute (FABI), Faculty of Natural and Agricultural Sciences, University of Pretoria, 0002 South Africa

**Keywords:** *Fusarium*, intron splicing, spliceosomal introns, *cis*-elements, gene prediction

## Abstract

Removal of introns from transcribed RNA represents a crucial step during the production of mRNA in eukaryotes. Available whole-genome sequences and expressed sequence tags (ESTs) have increased our knowledge of this process and revealed various commonalities among eukaryotes. However, certain aspects of intron structure and diversity are taxon-specific, which can complicate the accuracy of *in silico* gene prediction methods. Using core genes, we evaluated the distribution and architecture of *Fusarium circinatum* spliceosomal introns, and linked these characteristics to the accuracy of the predicted gene models of the genome of this fungus. We also evaluated intron distribution and architecture in *F. verticillioides*, *F. oxysporum*, and *F. graminearum*, and made comparisons with *F. circinatum*. Results indicated that *F. circinatum* and the three other *Fusarium* species have canonical 5′ and 3′ splice sites, but with subtle differences that are apparently not shared with those of other fungal genera. The polypyrimidine tract of *Fusarium* introns was also found to be highly divergent among species and genes. Furthermore, the conserved adenosine nucleoside required during the first step of splicing is contained within unique branch site motifs in certain *Fusarium* introns. Data generated here show that introns of *F. circinatum*, as well as *F. verticillioides*, *F. oxysporum*, and *F. graminearum*, are characterized by a number of unique features such as the CTHAH and ACCAT motifs of the branch site. Incorporation of such information into genome annotation software will undoubtedly improve the accuracy of gene prediction methods used for *Fusarium* species and related fungi.

Despite the increasing availability of whole-genome sequence information for diverse eukaryotes, genome annotation generally remains challenging (Yandell and Ence 2012). Apart from the computational complexities associated with the assembly of data generated by certain sequencing platforms, gene finding is particularly problematic (Loveland *et al.* 2012). This is mainly due to incomplete information on the inherent peculiarities associated with the structures of genes in different organisms, especially with regard to intron architecture, which severely limits optimization of *ab initio* gene prediction tools (*i.e.*, methods that use characteristic DNA sequences for genome annotations) for genome annotation (Ter-Hovhannisyan *et al.* 2008). Here the main issues are typically restrictions, not only in the availability of preexisting and accurate gene models, but also high-quality and appropriate reference genomes for accurately predicting intron–exon structures (Ter-Hovhannisyan *et al.* 2008).

The noncoding DNA sequences that interrupt the nuclear protein-coding genes of eukaryotes are referred to as spliceosomal introns (Bhattacharya *et al.* 2000). In contrast to group I and II introns with ribozymic activity (Bhattacharya *et al.* 2000), spliceosomal introns require the action of a large protein complex known as a spliceosome to facilitate their splicing (Jeffares *et al.* 2006). The length of spliceosomal introns typically varies depending on genome size and the level of expression of the genes that harbor them. Larger genomes generally tend to have longer introns (Deutsch and Long 1999), while highly expressed genes commonly harbor shorter introns (Castillo-Davis *et al.* 2002). The size of introns can also depend on their position within a gene. First introns, whether found in the 5′ untranslated region of a gene or in the open reading frame (ORF), are typically longer than those found downstream (Bradnam and Korf 2008). Furthermore, intron density varies widely in the genomes of eukaryotes. For example, the genomes of so-called “higher eukaryotes” such as human and *Arabidopsis thaliana*, contain ∼140,000 (Scott 1999) and 115,000 (Haas *et al.* 2005) introns, respectively. Those of “lower eukaryotes” such as fungi have fewer introns, *e.g.*, the fungal pathogen *Fusarium oxysporum* contains 34,137 introns (Croll and McDonald 2012), the yeast *Saccharomyces cerevisiae* has ∼253 introns (Scott 1999), and the microsporidian fungus *Encepalitozoon cuniculi* has only 15 introns (Scott 1999).

The functionality of spliceosomal introns is dependent on a number of intron-specific signature motifs or *cis*-elements to allow splicing (Deutsch and Long 1999). These *cis*-elements include the 5′ splice site, 3′ splice site, the branch site containing the branch point, and the polypyrimidine tract (Deutsch and Long 1999). Of these motifs, the polypyrimidine tract has been reported to be optional (Kupfer *et al.* 2004). During splicing, the components of the spliceosome (which consists of small nuclear ribonucleoprotein complexes) bind sequentially to the 5′ splice site, the branch site, and the 3′ splice site through RNA–DNA base pairing (Ast 2004; Russell 2006), after which the intron is excised and the two exons are ligated (Ast 2004; Russell 2006). However, splicing signals between closely related genomes can also be different (Kupfer *et al.* 2004), which is why most *ab initio* gene predictors lack high *cis*-element specificity. As a result, their ability to deal with introns that are apparently less conserved is severely impeded (Ter-Hovhannisyan *et al.* 2008).

In this study, we evaluated the accuracy of the automated gene predictions utilized to annotate the genome of the fungus *F. circinatum* (phylum Ascomycota, class Sordariomycetes, and order Hypocreales), which is the causal agent of the debilitating pitch canker disease of *Pinus* species (Wingfield *et al.* 2012). We also investigated the potential for refining existing gene prediction approaches in terms of intron distribution and architecture, as well as *cis*-element structure to allow improvements in the accuracy of the gene models for this fungus (Ter-Hovhannisyan *et al.* 2008). To achieve these goals, we compared the distribution and architecture of spliceosomal introns in this fungus with those occurring in the genomes of three other members of the genus, *i.e.*, *F. verticillioides* (a pathogen of maize), *F. oxysporum* (a pathogen of tomato), and *F. graminearum* (a wheat pathogen) [Leslie *et al.* 2006; *Fusarium* Comparative Database, Broad Institute (http://www.broadinstitute.org/annotation/genome/fusarium_group)]. Our specific aims were threefold: (i) to compile a dataset consisting of the sequences for the standard set of eukaryotic core genes (Parra *et al.* 2007) for the four fungi; (ii) to manually evaluate the external evidence (*e.g.*, EST data and protein alignments) for the automatically annotated introns; and (iii) to manually compare and contrast among the four fungi the distribution, length, phase, and *cis*-element structure of introns in the core gene set. Apart from providing information regarding the accuracy of the *F. circinatum* genome annotation and potential approaches to improve gene models, our results will significantly broaden current knowledge regarding the organization and sequence characteristics of fungal spliceosomal introns.

## Materials and Methods

### Fusarium circinatum cDNA library construction and sequencing

The *F. circinatum* EST data used in this study were obtained from two independent studies. In the first study, a pathogenic strain of *F. circinatum* (FSP34) was used to generate two cDNA libraries from carbon- and nitrogen-starved mycelia as described previously (Trail *et al.* 2003). Total RNA was extracted using TRI Reagent (Sigma) and Pure Zol RNA Isolation Reagent (Bio-Rad). The total RNA was purified using an RNeasy Mini Kit (QIAGEN), after which mRNA was isolated using an Oligotex mRNA Mini Kit (QIAGEN). Genomic DNA contamination was eliminated using On-Column DNase Digestion (QIAGEN) in combination with DNase I recombinant (Roche). The Roche cDNA Synthesis System was used to produce cDNA, which was then subjected to 454 pyrosequencing using the Roche 454 GS-FLX Titanium at Inqaba Biotec in South Africa.

In the second study, three *F. circinatum* strains (GL 57, GL 100, and GL 101) were used for the construction of two cDNA libraries on complete medium. One library included cDNAs from strains GL 57 and GL 100, and the other library cDNAs from strain GL 101. The libraries were generated by growing the *F. circinatum* isolates in potato dextrose broth (Difco laboratories) and extracting total RNA from the cultures using TRIzol (Invitrogen). Invitrogen’s Dynabeads mRNA Purification Kit was used to obtain mRNA. For cDNA synthesis, the Universal RiboClone cDNA Synthesis System (C4360; Promega) was used. Following purification using the MinElute PCR Purification Kit (QIAGEN), cDNAs were submitted for sequencing to the DNA Technology Facility at the University of California, Davis (U. C. Davis) using the Illumina GAIIx Sequencing platform.

### Fusarium genomes

The genome sequences for *F. graminearum*, *F. verticillioides*, and *F. oxysporum* were accessed from the Broad Institute’s *Fusarium* Comparative Database (http://www.broadinstitute.org/annotation/genome/fusarium_group), while the genome sequence for *F. circinatum* (accession number PRJNA41113) was available from a previous study (Wingfield *et al.* 2012). The *F. graminearum*, *F. verticillioides*, and *F. oxysporum* genomes have respective sizes of 36, 42, and 60 Mb (Ma *et al.* 2010), while that of *F. circinatum* is 44 Mb in size (Wingfield *et al.* 2012). Annotations of the *F. verticillioides*, *F. oxysporum*, and *F. graminearum* genomes have been done using GENEid (Guigo *et al.* 1992) and FGENESH (Solovyev *et al.* 1994) gene prediction software. The *F. circinatum* genome was annotated using MAKER (Cantarel *et al.* 2008), which incorporates the programs GeneMark-ES (Ter-Hovhannisyan *et al.* 2008), Augustus (Stanke and Waack 2003), and SNAP (Korf 2004).

### Identification of Fusarium core genes

The *Fusarium* core genes employed in this study corresponded to a set of 458 protein-coding core genes that are common to *S. cerevisiae* and presumably all eukaryotes (Holt and Yandell 2011). To compile the core gene set for *Fusarium*, the *S. cerevisiae* core genes were retrieved from the Ʃ-cegma (Core Eukaryotic Genes Mapping Approach; Parra *et al.* 2007) website (http://korflab.ucdavis.edu/Datasets/cegma/Appendix.html) and used in BLASTp searches against the *Fusarium* Comparative Database at the Broad Institute. FASTA files were subsequently compiled with the *F. verticillioides*, *F. oxysporum*, and *F. graminearum* nucleotide sequences for the core genes. The *F. verticillioides*, and in a few cases the *F. oxysporum* and *F. graminearum*, core gene sequences were then used to identify and retrieve the corresponding sequences from the *F. circinatum* genome by making use of BLASTn, CLC Genomics Workbench version 3.7.1 (CLC bio A/S), and BioEdit version 7.0.9.0 (Hall 1999). In all cases, default parameters were used for the BLAST searches.

### Identification and annotation of introns

Alignments of the core genes of the four *Fusarium* species were performed in BioEdit using the ClustalW Multiple Alignment tool (Thompson *et al.* 1994). Predicted intron positions in the core genes of *F. verticillioides*, *F. oxysporum*, and *F. graminearum* were assessed using the Broad Institute gene models. Intron positions in the *F. circinatum* core genes were assessed by employing the MAKER gff annotations and the Apollo Genome Annotation Curation Tool (Lewis *et al.* 2002). The identified introns were then annotated in CLC Main Workbench 5.7 (CLC bio A/S).

ClustalW alignments of all core genes with apparently incongruent (nonconserved) intron positions were analyzed in Augustus version 2.4 (Stanke and Waack 2003; Stanke *et al.* 2008). This computer software predicts gene elements by using intrinsic *ab initio* algorithms and extrinsic experimental data such as EST sequences. Relevant EST data for *F. verticillioides*, *F. oxysporum*, and *F. graminearum* was available from the Broad Institute, while those for *F. circinatum* were obtained from the Sequence Read Archive (SRA; accession number SRR1168456) of the National Center for Biotechnology Information (NCBI; http://www.ncbi.nlm.nih.gov/sra). We also included the EST data for two additional strains of *F. circinatum* used in an ongoing study (S. L. Slinski and T. R. Gordon, unpublished data). The *F. graminearum* default transition matrix (*i.e.*, the matrix of transition probabilities for predicted gene models) was used because it is the only *Fusarium* species for which a matrix is available in Augustus. In addition to resolving incongruent intron positions, the EST data of the four genomes was also useful for identifying unusual *cis*-elements (see below).

### Analysis of intron frequency, length, and distribution

For the analysis of intron length, frequency, and distribution, a subset of core genes was used. This subset included 226 core gene sequence alignments that were arbitrarily selected. Each alignment contained one gene from each of the four *Fusarium* species. Any gene that showed intron position incongruence among the four *Fusarium* species without EST data support was excluded from this dataset.

The dependence of intron length on its position in the coding sequences (CDS) of a gene was tested using ANOVA (Samuels and Witmer 2003) and hypothesis testing was done using the *F* test where the null (*H*_o_) hypothesis was that all the mean lengths of the introns in different positions are equal (Samuels and Witmer 2003). For this purpose, the *F* statistic was compared to an *F* distribution critical value at a 99.99% confidence level (*P* = probability = 0.001) with 6 (numerator) and 500 (denominator; the exact denominator values for *F. verticillioides*, *F. circinatum*, *F. oxysporum*, and *F. graminearum* were 497, 498, 498, and 496, respectively) d.f. (Samuels and Witmer 2003). To test whether the observed differences between means were significant, Tukey’s Honestly Significant Difference (HSD) test was used. For this purpose, the equation *q* was used (*q* is Tukey’s statistic, *M*_1_ and *M*_2_ are the two means being tested, *MS_w_* is the mean of squares within the data, and *n* is the number of samples per treatment group). Critical values for Tukey’s HSD (*q*) were determined using the d.f. (∞; the exact values for *F. verticillioides*, *F. circinatum*, *F. oxysporum*, and *F. graminearum* were 497, 498, 498, and 496, respectively) and the number of treatments (7), which is the number of intron positions compared.

For the analyses of relationships between gene length and intron length, and gene length and number of introns per gene, scatter plots were generated using Microsoft Excel 2010. The significance of regression lines were tested using the Student’s *t*-test, where *H*_o_ and *H*_a_ were, respectively β_1_ = 0 and β_1_ ≠ 0 (β_1_ is the slope of the regression line) (Samuels and Witmer 2003). When β_1_ = 0, there is no correlation between gene length and intron length, or gene length and number of introns per gene, and the null hypothesis will be accepted. The *t* statistic was obtained with the equation *t_s_* = *b*_1_/SE*_b_*_1_, where *t_s_* is the *t* statistic, *b*_1_ is the slope of the regression line, and SE*_b_*_1_ is the SE of the slope. For these calculations, the Excel Regression Tool found in the Excel Analysis ToolPack and Excel Analysis ToolPack-VBA Add-ins (Arthur 2012) were used. The *t* critical values were obtained from the Student’s *t* distribution table at a probability value of *P* = 0.10 and 0.05 with *n* − 2 d.f. (Samuels and Witmer 2003).

### Analysis of intron phase

The 226 core gene dataset described above was used to analyze the intron phase. Intron phases were examined manually in BioEdit (Hall 1999), using the CDSs together with the protein and genomic sequences. The “Toggle Translation” function in BioEdit was used to determine whether introns were in phase 0, phase 1, or phase 2.

### Identification and analysis of intron cis-elements

The three main *cis*-elements (5′ splice site, branch site with the branch point, and 3′ splice site) of introns from all the genes in the dataset were examined. To achieve this, the annotated introns were extracted from each core gene alignment using the “Extract Sequences” and then the “Extract Annotations” functions in the CLC Genomics Workbench. In all cases, an additional five nucleotides in the upstream exon and five additional nucleotides in the downstream exon were extracted with the introns. The extracted introns were then aligned using the “Create Alignment” function after which they were manually annotated relative to the annotations previously produced for fungal introns (Kupfer *et al.* 2004; Bhasi *et al.* 2007). A preliminary *cis*-elements motif list was constructed using the “Create Motif List” function. For this purpose, a subset of 100 introns from the core genes were considered. From these, preliminary 5′ and 3′ splice site and branch site motifs were generated by manually obtaining motifs of these *cis*-elements from previous fungal gene annotations. A list of the motifs was then generated and used as a guide to search for motifs specific to the core genes of the four *Fusarium* species that were studied. When unique motifs were found, they were added to the motif list. The motif list was then used to perform a motif search on the rest of the gene sequence alignments, and sequence variants of these elements were continuously added when new motifs were encountered.

To examine the structure of the intron polypyrimidine tracts in *Fusarium*, the introns of 10 randomly selected genes were used (only these were used because of the high variation observed in position, length, and potential number of polypyrimidine tracts per intron). A minimal definition of six consecutive nucleotides with at least three 2′-deoxythymidines (*i.e.*, uridines in the transcribed sequence) and no 2′-deoxyadenosine was used for this purpose (Kupfer *et al.* 2004). The polypyrimidine tracts were analyzed using the sequence, position, and intron region occupied by the tract (*i.e.*, the polypyrimidine tract may occupy the region between the 5′ splice site and the branch site or the region between the branch site and the 3′ splice site) (Kupfer *et al.* 2004).

### Data availability

All the genome sequences and EST data used in this study are available without restriction. *F. circinatum* genome sequence accession number: PRJNA41113. *F. circinatum* EST data accession number: SRR1168456. The *F. graminearum*, *F. verticillioides*, and *F. oxysporum* data were accessed from the Broad Institute’s *Fusarium* Comparative Database (http://www.broadinstitute.org/annotation/genome/fusarium_group) and are currently available from the Broad Institute fungal ftp site: ftp://ftp.broadinstitute.org/pub/annotation/fungi/fusarium.

## Results

### Identification of a set of core genes

The use of a set of 458 core genes common to all eukaryotes (Parra *et al.* 2007) produced 458 significant BLASTp hits from the *Fusarium* Comparative Database at the Broad Institute. We intended to use the identified *F. verticillioides* genes in BLASTn searches to identify homologs in the genome of *F. circinatum*. However, homologs for 12 of the 458 genes were absent in the *F. verticillioides* genome and, to compensate for these, we instead used genes from *F. graminearum* (nine) and *F. oxysporum* (three). These searches allowed for the identification of the full set of 458 core gene homologs in the *F. circinatum* genome, although 22 were separated over multiple contigs and thus excluded from further analyses. The final core gene dataset utilized in this study thus consisted of 436 core gene sequences.

### Evaluation of external evidence for predicted introns

Evaluation of the external evidence for the introns predicted in the set of core genes utilized in this study was based on EST data and comparisons of intron positions using multiple alignments. However, due to lack of availability, we generated EST data for four isolates of *F. circinatum*. The genome sequence for one of these (*i.e.*, FSP34) has been determined previously (Wingfield *et al.* 2012). The other three isolates (GL 57, GL 100, and GL 101) originated from a doctoral study at U. C. Davis (Unplublished data). A total of 61,000 reads were generated for FSP34 using the 454 platform, while 16,118 reads were generated from the combined libraries of isolates GL 57, GL 100, and GL 101 using the Illumina platform. Following quality filtering, assembly of these reads generated 470 and 184 contigs, respectively. The ESTs for FSP34 are available at the NCBI SRA (accession number SRR1168456), while those for isolates GL 57, GL 100, and GL 101 are available from S. L. Slinski and T. R. Gordon (U.C. Davis).

Of the 436 core genes examined, 152 appeared to be nonconserved with respect to intron position. To ensure that this lack of conservation was not due to the different gene prediction methods used during annotation of the four genomes, all 152 genes were reanalyzed with Augustus (Stanke and Waack 2003; Supplemental Material, File S1) using the available EST data. The analysis revealed that 54% (82) of the 152 genes were wrongly annotated. In part, this was due to the fact that dissimilar gene prediction software was used for the four *Fusarium* genomes. Also, Augustus does not recognize GC–AG introns as was previously also shown for *Armillaria mellea* (Misiek and Hoffmeister 2008). The reanalysis showed that these genes actually harbored positionally conserved introns that correlated with the positions in the genomes of at least one of the three other species. Because we conducted all these analyses during 2012 and the gene models for the various *Fusarium* genomes could have been improved since then, we repeated our analyses in February 2014 as well as in October 2015. After reanalyzing the gene models in 2014, we found that 18 of the original 82 wrongly annotated gene models had been updated since 2012, and that these are now in agreement with ours. After the 2015 reanalysis, we found that another three models were updated, which are now also in agreement with ours. In addition to the intron position discrepancies, we found that some gene models (∼2% of the *F. ciricinatum* core genes) erroneously had very short introns of 5–23 nucleotides (nt) in length, which were subsequently resolved using the EST data. After this process of EST-based position resolution, introns with nonconserved positions were present only in 69 of the 436 core gene alignments. Therefore, the large majority (84%) of the core genes examined harbored introns with conserved positions.

For the 69 genes with introns that were apparently not conserved with respect to position, EST data were available for 23 genes only. Examination of the 46 gene alignments lacking EST support revealed five genes for which positional incongruences among introns were due to nucleotide substitutions. In one case, *F. graminearum* had AT instead of the canonical 5′ splice site GT at the beginning of the intron, as was observed for the other three *Fusarium* species. In another case, *F. graminearum* had TT instead of GT at the 5′ splice site, and in two other instances had GC at this site (underlined nucleotides represent substitutions). *F. oxysporum* had one case of a GT to GA substitution. Seventeen gene alignments had nonconserved intron positions due to intron insertions/deletions (indels) (seven from *F. graminearum* and *F. oxysporum* each, and three from *F. verticillioides*). A number of gene alignments had shorter (four in *F. graminearum*, two in each of *F. verticillioides* and *F. oxysporum*, and three in *F. circinatum*) or longer predicted ORFs (two in *F. circinatum*). Eleven gene alignments contained at least one truncated (*i.e.*, not fully sequenced) homolog of the gene in one of the species examined. All the above-mentioned gene alignments were excluded from the core gene dataset used in subsequent analyses.

For the remaining 23 genes for which intron positions were supported by EST data, the apparent lack of positional conservation was due to one of three factors. Of the 23 genes, 15 from *F. graminearum* had whole-intron deletions, while one had a 2′-deoxythymidine to 2′-deoxyguanosine (T > G) substitution in the second nucleotide of the 5′ splice site. Of the six intron position incongruences observed in *F. oxysporum*, three were as a result of whole-intron deletions, one had a shorter ORF, while two were divergent in nucleotide sequence when compared to the other *Fusarium* species. Of the three genes with nonconserved intron positions in *F. circinatum*, one gene had a whole-intron deletion and two had shorter ORFs compared to the other three species.

### Comparison of intron characteristics

#### Intron frequency, length, and distribution:

**Figure 1:**
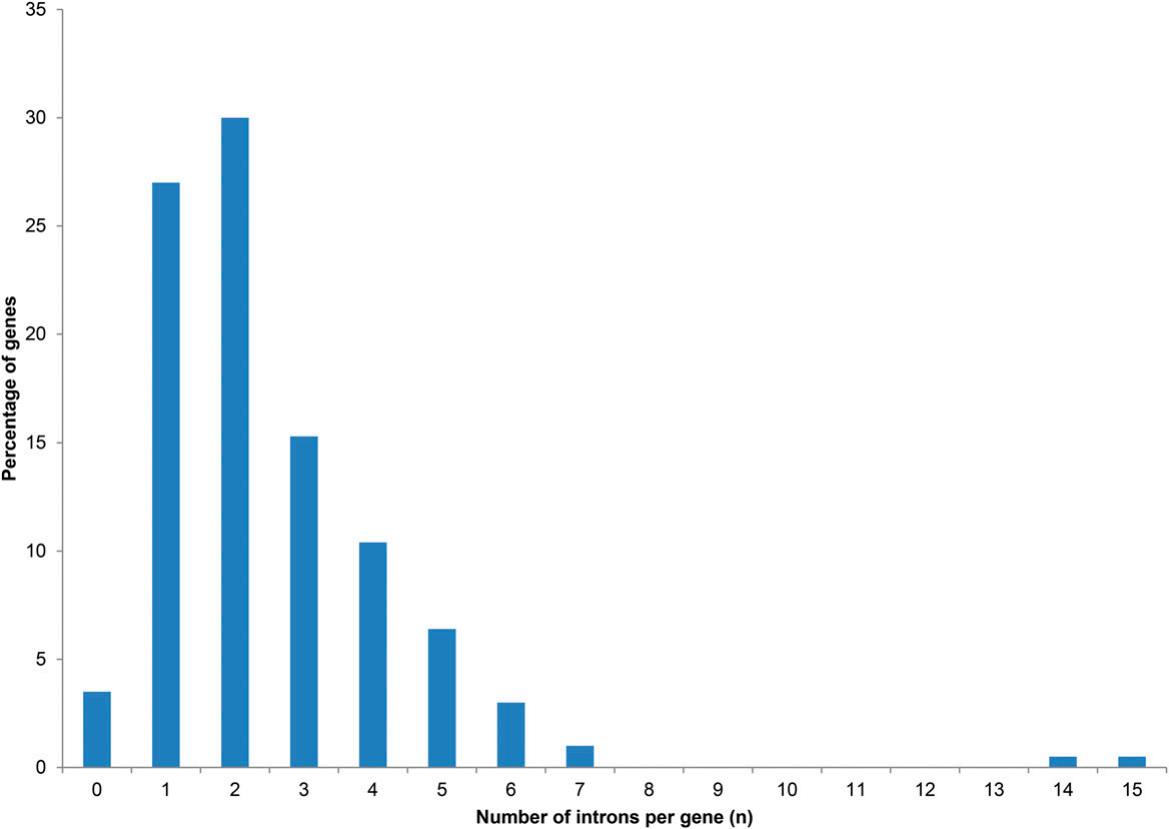
Frequency of introns in the coding sequences of 226 core genes in four *Fusarium* species.

For the analysis of intron length, frequency and distribution, a subset of the CDSs for 226 core genes were used. Our initial dataset consisted of ∼436 genes for each of the four *Fusarium* species we examined. We made alignments and manually annotated all the genes. However, for manual in-depth analyses we scaled the number of genes down to a manageable set of 226 genes for each species. Within each of these datasets, the number of introns ranged from 0 to 15 introns per CDS (Figure 1; also Table S1 in File S2 of the supplementary material for a list of genes and their number of introns identified), with an average density of 2.53 introns per CDS. However, consistent with what has been observed for *Neurospora crassa* (Bruchez *et al.* 1993), no significant relationship was found between CDS length and the number of introns per CDS in the four *Fusarium* species (see Figure S1 in File S3). Within this dataset, the gene encoding CTP synthetase had the highest number of introns (15 in all four *Fusarium* species), followed by that encoding glutamine-dependent NAD^+^ synthetase with 14 introns in *F. verticillioides*, *F. circinatum*, and *F. oxysporum*, and 12 in *F. graminearum* (also see Table S1 in File S2). Of the 226 examined core genes, seven harbored no introns in any of the four *Fusarium* species and these included genes encoding pre-mRNA splicing factor clf, amino methyltransferase-mitochondrial precursor, seryl tRNA synthetase, DNA pantothenate metabolism flavoprotein 2, Sol1 family protein, uridylate kinase, and the DNA repair helicase RAD25.

**Figure 2:**
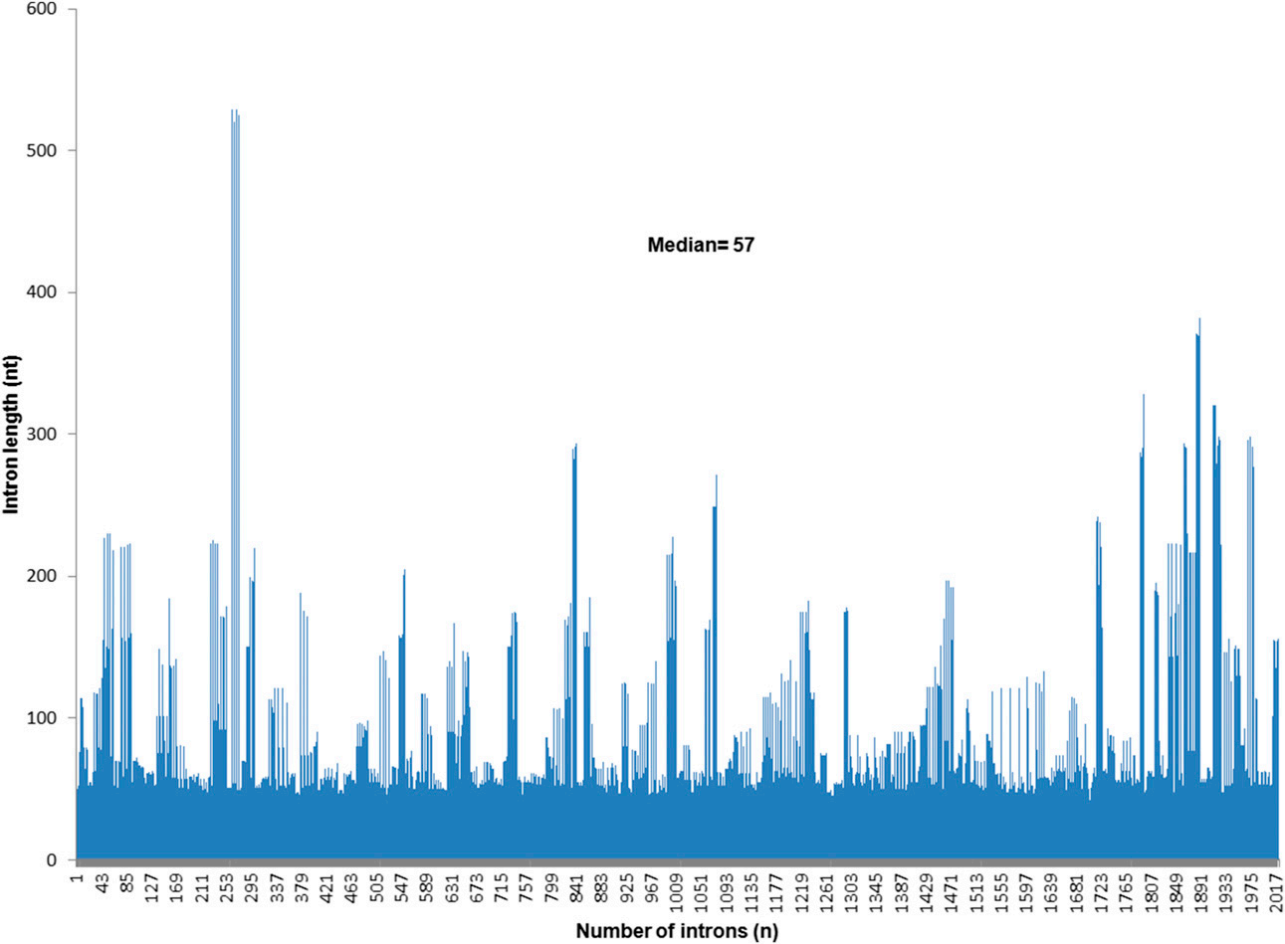
Lengths of all introns within a set of 226 coding sequences of four species of *Fusarium*.

Among the 226 CDSs examined, intron length in the four *Fusarium* species was on average 75.4 nt and ranged from 42 to 529 nt with a median of 57 nt (Figure 2). The mean intron lengths for *F. verticillioides*, *F. circinatum*, *F. oxysporum*, and *F. graminearum* were, respectively, 75.8 (44–529), 76.3 (42–520), 75.8 (42–529), and 75.7 (43–525) nt, where the numbers in parentheses denote intron size range. In almost all alignments, the lengths of introns in the core sequences of *F. graminearum* were different from those of *F. verticillioides*, *F. circinatum*, and *F. oxysporum* (see Table S2 in File S4).

**Figure 3:**
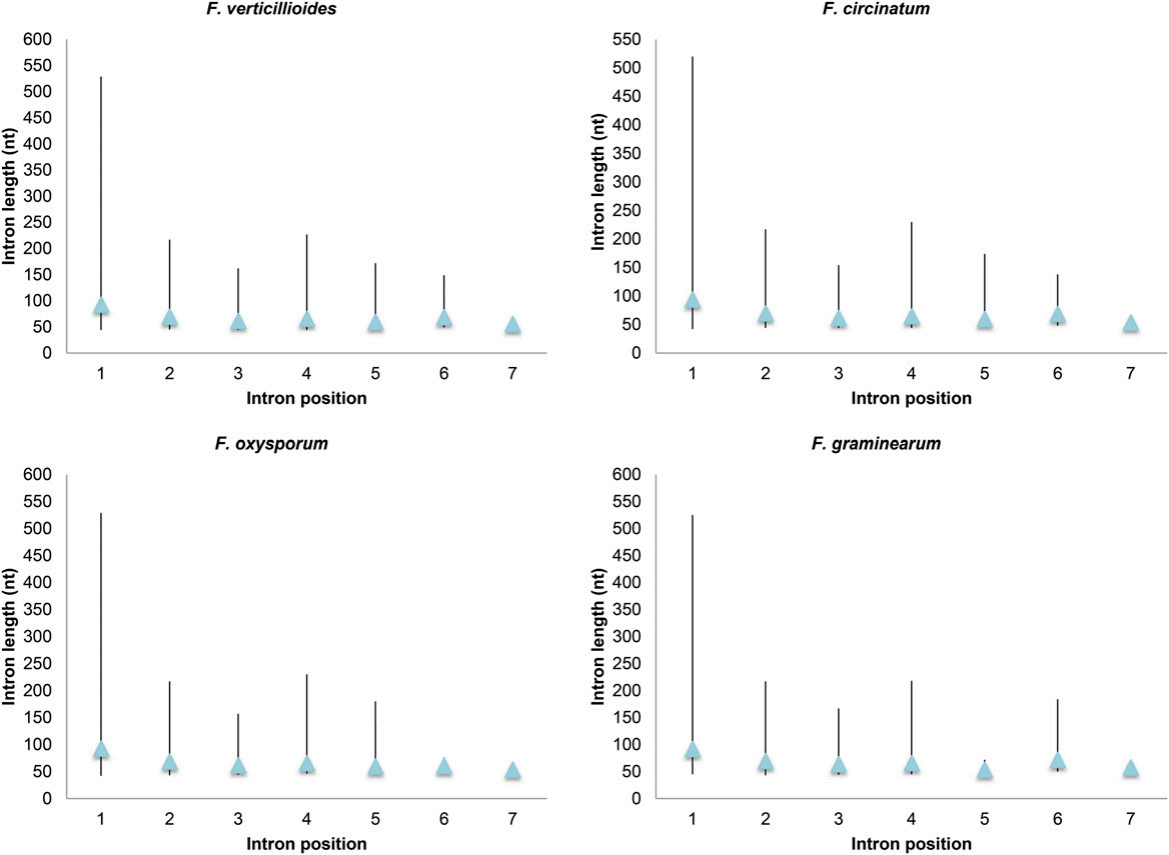
The relationship between intron position and intron length in 226 core genes of four *Fusarium* species. The graphs were plotted with three data points (high, low, and mean intron lengths) on the *y*-axes for the intron positions on the *x*-axes. The vertical lines represent the high and low intron lengths and the blue triangles represent the mean values. An ANOVA and Tukey’s Honestly Significant Difference tests showed that the mean lengths for the first-position introns were significantly different from introns in positions 2–7 (*P* = 0.05).

Longer introns were mostly located at the 5′ end of CDSs. This phenomenon was more pronounced when first introns were compared with the rest of the introns in the core dataset. The mean intron length of the first introns from all four *Fusarium* species was ∼93 nt (42–529). The rest of the introns at positions 2–7 had mean intron lengths between 54 and 72 nt (introns in positions 8–15 were excluded due to inadequate data for statistical analyses). A similar trend was observed for all four species when analyzed independently (Figure 3). Based on ANOVA, *F* statistic values of 6.277, 6.801, 6.374, and 6.027 were obtained for *F. verticillioides*, *F. circinatum*, *F. oxysporum*, and *F. graminearum*, respectively. The *F* distribution critical value for all the species was 3.81, which showed that the mean intron lengths were significantly different. For all species, the null hypothesis that the mean intron lengths at all CDS positions are equal was thus rejected at *P* = 0.001. The results of Tukey’s HSD tests further indicated that the mean sizes of introns in first CDS positions were significantly different from those for introns in downstream positions. Values for Tukey’s statistic for *F. verticillioides*, *F. circinatum*, *F. oxysporum*, and *F. graminearum*, when *M*_1_ (mean size of first introns) was compared to *M*_2_ through to *M*_7_ (mean intron sizes at positions 2–7, respectively) in a pairwise manner, were found to be greater than 6.67, 6.73, 6.73, and 5.71, respectively. All other comparisons that excluded the mean sizes of the first introns produced values < 1.94. The critical value at *P* = 0.05 was 4.17, indicating that the null hypotheses were only rejected when *M*_1_ was compared with *M*_2_ to *M*_7_.

A statistically significant negative correlation between CDS length and intron length was observed, where longer genes had shorter introns (Figure S2 in File S3). The Student’s *t* statistic values for *F. verticillioides*, *F. circinatum*, *F. oxysporum*, and *F. graminearum* were, respectively, 1.67, 1.98, 1.62, and 1.90. With *P* values of 0.10 and 0.05, and *n* − 2 d.f., the *t* critical values were 1.65 and 1.96, respectively. The null hypothesis that there is no correlation between CDS and intron length was rejected for *F. verticillioides* and *F. graminearum* at the 90% confidence level, and for *F. circinatum* at both confidence levels. Only in the case of *F. oxysporum* could the null hypothesis not be rejected at either confidence level because of the low *t* statistic values.

**Figure 4:**
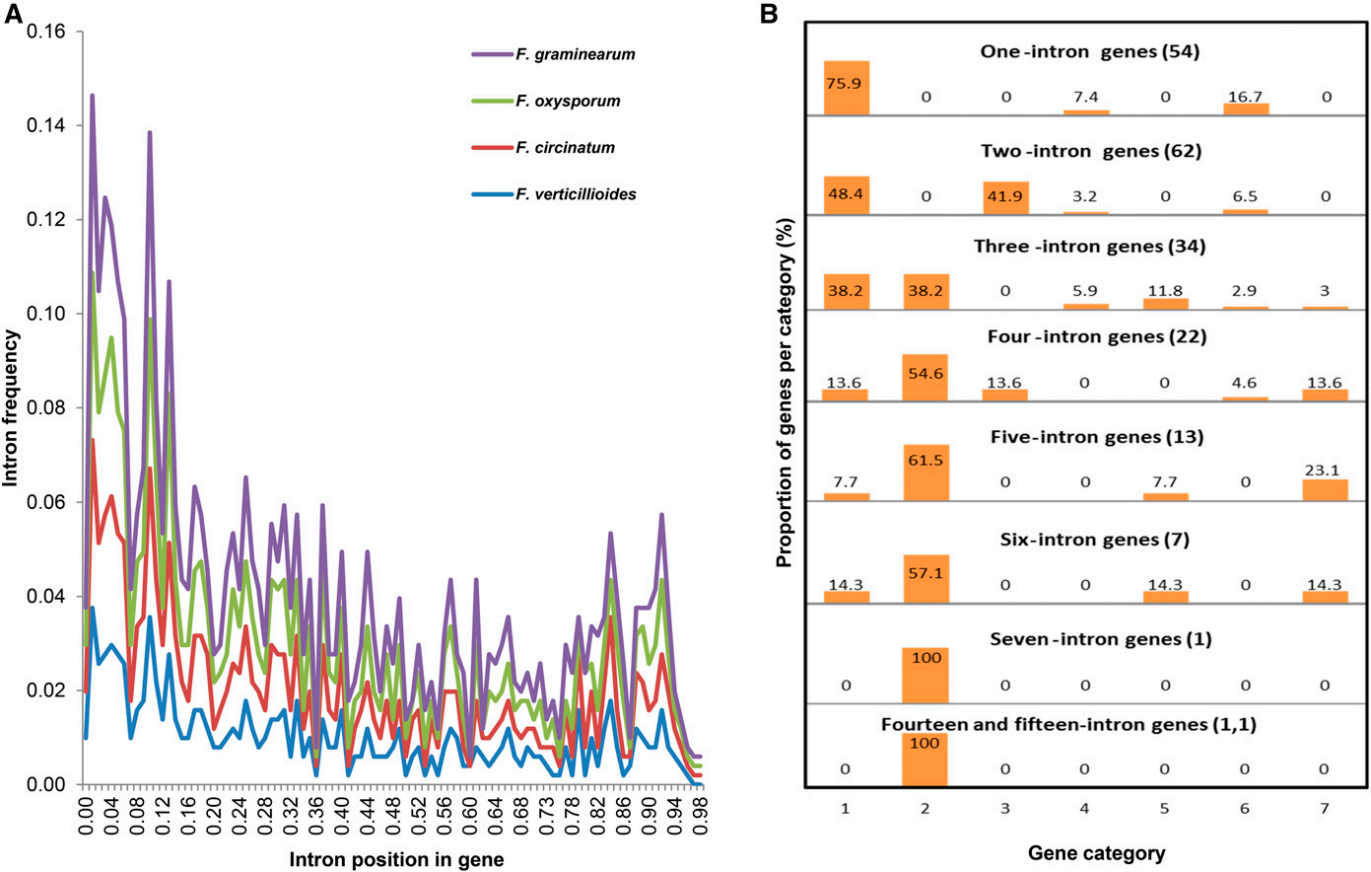
The distribution of introns within the set of 226 core genes of the four *Fusarium* species. (A) The positions of introns are shown along relative gene length (*x*-axis), and the frequencies of these introns are depicted on the *y*-axis. (B) The genes were divided into three regions: the 5′ region [the first third of the coding sequence (CDS)], the middle region (the second third of the CDS), and the 3′ region (the last third of the CDS). Gene categories: 1, all introns are at 5′ region; 2, > 50% of the introns are in the 5′ region; 3, 50% of the introns are in the 5′ region and 50% are in the 3′ region; 4, all introns are in the middle region; 5, > 50% of the introns are in the 3′ region; 6, all introns are in the 3′ region; and 7, introns are evenly distributed across the gene (no concentration of introns at a particular region). The numbers in parentheses are the number of CDSs included per gene category.

Analysis of the distribution of introns within the CDSs of the 226 core genes revealed a 5′ region positional bias (Figure 4A). The largest proportion of the genes examined showed this intron positional bias, where either all introns were located in the first third of a CDS or > 50% of its introns were located in this region (Figure 4B). This bias was seen for genes harboring 1–15 introns. Only in the case of two- and four-intron genes did equal numbers of introns occur in the first and last third of a CDS (*i.e.*, in the 5′ and 3′ region). In a few cases all introns were located in the middle of CDSs, in few others there was a 3′ positional bias within the CDS, and in a limited number of instances (*i.e.*, four-, five-, and six-intron genes) there was an approximately even distribution of introns across the CDS. The distribution of introns in the CDSs of the four *Fusarium* species followed the same trend when they were analyzed separately.

#### Intron phase:

Our analysis showed that introns of phases 0 (when an intron is located between two codons), 1 (an intron that is located between the first two nucleotides of a codon), and 2 (when an intron is located between the last two nucleotides of a codon) were present in all four *Fusarium* species, albeit at different frequencies. In the set of CDSs for 226 core genes, most introns were phase 0, which appeared to be true also for the one, three-, four-, five-, seven-, eight-, nine-, and twelve-intron genes (see Figure S3 in File S3). Although the number of introns in phase 0 was lower than the number of phase 1 and phase 2 introns added together, the number of phase 0 introns was the highest when all three phases were compared individually. In addition, the 5′ regions of CDSs contained more phase 0 introns than phase 1 and 2 introns (see Figure S3A in File S3). The highest proportion of introns in the first and the fourth quarter of the genes were in phase 0. Although negligible, this was also the case for introns in the second quarter of the genes (see Figure S3B in File S3).

The respective ratios of the three intron phases (0:1:2) in the four fungi were 215:172:147 for the 534 examined introns of *F. verticillioides*, 212:171:143 for the 526 introns examined in *F. circinatum*, 212:171:141 for the 524 introns of *F. oxysporum*, and 211:172:137 for the 520 introns in *F. graminearum*. For genes with the same number of introns, the phases were conserved among the four *Fusarium* species. The only two exceptions were in the CDS of the gene encoding glutamine-dependent NAD + synthetase in *F. graminearum*, where the eighth intron was phase 1 and not phase 0, and the fourteenth intron was phase 0 and not phase 2, in comparison to the other *Fusarium* species.

Our data further showed that the analysis of intron phase could potentially aid in the identification of potential sequencing errors. This was primarily in terms of errors associated with mononucleotide tracts. In four instances [two in *F. circinatum* (superoxide dismutase mitochondrial precursor and heat shock 70 kDa protein), and one each in *F. oxysporum* (protein phosphatase PP2A regulatory subunit A) and *F. graminearum* (glucose 6 phosphate 1 dehydrogenase)], each of the sequences had an extra nucleotide that, when deleted, generated an intron phase similar to those in the other species. Another potential sequencing error involving a pyrimidine substitution was observed in *F. circinatum* (AP 2 adaptor complex subunit α).

#### Intron cis-elements:

**Figure 5:**
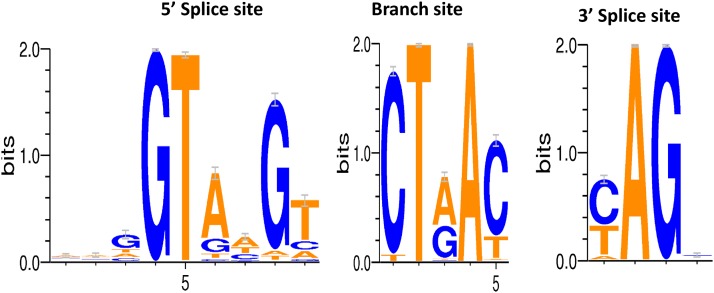
Consensus sequences at the 5′ splice site, the branch site, and the 3′ splice site constructed using WebLogo 3.3. bits, binary digits.

For the 226 core gene dataset, alignments of the 5′ splice site were performed for the 2022 introns identified. From these, the following consensus motif was obtained: A_38_A_38_G_53_|G_100_T_99_A_74_A_42_G_93_T_66_ (Figure 5 and Table 1). Each of the four *Fusarium* species had the above consensus motif when analyzed separately. Here, the subscripts denote the percentage conservation of a base at a particular position and the vertical bar represents the exon–intron junction. The raw sequences of each species are provided in File S4.

**Table 1:** The length of introns and a summary of the motifs examined in 2022 introns from 226 core genes in four *Fusarium* species

Length*^a^*	5′ Splice Site Motif*^b^*	Polypyrimidine Tract	Branch Site Motif*^c^*	3′ Splice Site Motif*^d^*
42–529 nucleotides	A_38_A_38_G_53_|G_100_T_99_A_74_A_42_G_93_T_66_	83% located between 5′ splice site and the branch site, 17% located between the branch site and the 3′ splice site	CTRAY (91%)	Y_93_A_100_G_100_ |R_59_
			CTHAH (4.99%)	YAG|R (94.31%)
			TTRAY (3.96%)	RAG|R (3.51%)
			ACCAT (0.05%)	RAG|Y (2.18%)

a Subscript digits following individual bases indicate the proportion (in percentage) of occurrence of the base in that position.

b For comparison with the literature, we included the 5′ splice site consensus sequence in this form. However, full details regarding the frequency of specific bases are as follows: N^1^N^2^N^3^|G_100_YN^4^N^5^N^6^N^7^, where N^1^ = A_38_/G_21_/T_19_/C_24_; N^2^ = A_38_/G_20_/T_21_/C_21_; N^3^ = A_15_/G_53_/T_15_/C_1_; N^3^ = A_15_/G_53_/T_15_/C_17_; N^4^ = A_74_/G_16_/T_6_/C_4_; N^5^ = A_42_/G_4_/T_22_/C_32_; N^6^ = A_3_/G_93_/T_3_/C_1_; N^7^ = A_14_/G_4_/T_66_/C_16_; and Y = T_99_/C_1._

c The proportion of the introns in which a specific branch site motif was observed is indicated in parentheses. Alternative branch site sequences: CTHAH represents CTTAC, CTCAA, CTAAA, and CTCAT; TTRAY represents TTAAC, TTAAT, TTGAC, and TTGAT*. All the predicted branch site motifs were supported by expressed sequence tags data, except for TTGAT. Within the sequences, R, H, and Y represent standard International Union of Pure and Applied Chemistry codes for degenerate nucleotides, where R represents a nucleotide with either 2′-deoxyguanosine or 2′-deoxyadenosine bases, Y represents either 2′-deoxycytidine or 2′-deoxythymidine bases, and H represents 2′-deoxyadenosine, 2′-deoxycytidine, or 2′-deoxythymidine bases.

d The proportion of the introns in which a specific 3′ splice site was observed is indicated in parentheses.

Analysis of the 2022 introns also allowed refinement of the 3′ splice and branch site motifs (Figure 5 and Table 1). Alignments of these introns generated a well-resolved 3′ splice site motif consensus with the sequence Y_93_A_100_G_100_|R_59_ (Y and R denote nucleotides with pyrimidine and purine bases, respectively). However, the presence of two variants (RAG|Y or RAG|R) in a small proportion of the introns (115, 5.69%) examined was also revealed (File S4). With regard to the branch site motif, > 90% of the sites had the consensus CTRAY, while three additional variants (TTRAY, CTHAH, and ATCAT; H denotes a nucleotide with a 2′-deoxyadenosine, 2′-deoxycytidine, or 2′-deoxythymidine base) were also detected in a small number of introns. The same motifs were observed for the each of the four *Fusarium* species.

The polypyrimidine tract (a minimum of six consecutive nucleotides with at least three 2′-deoxythymidines and no 2′-deoxyadenosines; Kupfer *et al.* 2004) was diverse among the introns of the four *Fusarium* species (File S4). In a dataset containing 166 introns from 10 genes, the length of the predicted polypyrimidine tracts ranged from 6 to 25 nt. The number of predicted polypyrimidine tracts per intron also varied considerably. In some cases, as many as nine were predicted, while in 24 instances no sequence resembling this element could be detected. For more than half (53%) of the introns analyzed either one or two polypyrimidine tracts could be predicted. Our analyses also showed that the predicted polypyrimidine tracts occupied two intron regions, the 5′ region and the 3′ region, where the branch site was the reference point (Kupfer *et al.* 2004). In all four *Fusarium* species, the majority of the predicted polypyrimidine tracts occupied the 5′ region (*i.e.*, 8–40 nt away from the 5′ splice site). Only 14–20% of the predicted polypyrimidine tracts occupied the 3′ region. Of the latter, *F. graminearum* had the most predicted polypyrimidine tracts in the 3′ region, while *F. oxysporum* had the fewest. In total, 61% of the examined introns had predicted polypyrimidine tracts exclusively in their 5′ region, 35% exclusively in their 3′ region, and 5% in both the 5′ and 3′ regions (Table S3 in File S2).

## Discussion

### Manual curation increases the accuracy of genome annotations

The results of this study emphasize that manual curation represents a critical aspect of the annotation of any genome. By making use of a set of core genes and manually evaluating their intron–exon boundary predictions, we produced highly defined consensus sequences for the splice site junctions and internal *cis*-elements of spliceosomal introns for *F. verticillioides*, *F. oxysporum*, and *F. graminearum*. In other words, manual curation of automatically annotated gene models, together with the use of external evidence for these models, would significantly increase the quality of a genome annotation (Loveland *et al.* 2012). For *Fusarium*, this was clear from the apparent improvements observed (even with our small subset of the *Fusarium* gene complement) in gene models involving nonconserved intron positions during the 2012–2015 period. A similar scenario was observed for *F. graminearum*. During their study of alternative intron splicing in this fungus, Zhao *et al.* (2013) found that a large number of gene models in the Broad Institute *Fusarium* Comparative Database are incorrect. In a subsequent study (King *et al.* 2015), reannotation using improved contemporary algorithms and manual curation significantly increased the quality of its genome annotation. These findings, together with those presented in the current study, thus highlight the value and importance of EST-based validation of gene models, even when closely related species are examined. Although predictions remain subject to laboratory experimentation for confirmation, their initial manual curation and evaluation can go a long way toward at least validating them *in silico*.

### Intron length and density in Fusarium

The mean lengths for the introns in the core genes of the four *Fusarium* species are shorter than those reported as a general average for fungal spliceosomal introns. The latter has been suggested to be ∼85 nt in length (Hawkin 1988), which is comparable to the 83 nt mean intron length reported for *F. graminearum* when the whole genome of this species was examined (Wong *et al.* 2011). In our set of core genes, which included those for *F. graminearum*, introns were on average 76 nt long. This difference probably reflects the fact that constitutively expressed genes, such as most of the core genes, generally have shorter introns to facilitate more efficient splicing (Comeron and Kreitman 2000; Hurst *et al.* 1999). It is thought that natural selection acts to retain shorter introns or purge longer introns (Castillo-Davis *et al.* 2002). In fact, with a eukaryotic transcription rate of 20 nt/sec (Ucker and Yamamoto 1984; Izban and Luse 1992) at an energy cost of two ATP/nt (Lehninger 1975), shorter introns would allow for much shorter processing times and more efficient or rapid expression of the genes in which they occur. However, strong evidence against this energetic cost hypothesis for introns in highly expressed genes has been presented (Huang and Niu 2008). The exact reasons for the correlation between intron size and gene expression levels therefore remain unclear.

Compared to other eukaryotes, the genes of *Fusarium* species and other fungi are characterized by relatively low spliceosomal intron densities. A recent study conducted on whole genomes of publicly available *Fusarium* species revealed that *F. oxysporum* has an average of 1.9 introns per gene, whereas an average of 1.8 introns per gene was reported for *F. graminearum* (Croll and McDonald 2012). This is slightly higher than the 1.5 introns per gene that were found for other fungi (Jeffares *et al.* 2006). In the present study, the average intron density per core gene for the *Fusarium* species was determined at 2.53 introns per gene. In contrast, studies on *S*. *cerevisiae* have shown that essential genes, such as ribosomal protein genes, have fewer introns (Parenteau *et al.* 2011). The differences among the studies on the various filamentous fungi could in part be explained by the differences in gene sets compared. However, relative to *S*. *cerevisiae* (Parenteau *et al.* 2011) and other fungi (Jeffares *et al.* 2006), our findings suggest that core genes, at least in *Fusarium* species, are more likely to retain introns during evolution.

The results of this study, together with those of earlier studies, indicate that the intron densities in fungi are lower than those reported for plants and animals (Scott 1999; Haas *et al.* 2005). According to Jeffares *et al.* (2006), the relatively low intron densities in fungi correlate with their low complexity and short generation time. For example, *A. thaliana* and human, with their considerably longer generation times, have 4.3 and 8.82 introns per gene, respectively (Jeffares *et al.* 2006). Also, the complexity of plants and animals has been linked to the extra levels of gene expression regulation, where introns influence transcription initiation, pre-mRNA polyadenylation, mRNA decay, mRNA transport, and translation (Le Hir *et al.* 2003). Information regarding the contribution of intron-mediated expression regulation in fungi is limited and future studies should seek to determine whether the relatively low intron density in fungi means that intron-mediated expression regulation is not common in these organisms.

### Intron position is occasionally linked to intron length, conservation, and phase

Consistent with what has been found in other eukaryotes (Bradnam and Korf 2008), the first introns of the *Fusarium* core genes were generally longer than those occurring in downstream positions. First introns are mostly at the 5′ end of genes and could represent “early” or ancestral introns (Nguyen *et al.* 2006), which have had adequate time to accumulate extra (“junk”) DNA (Bradnam and Korf 2008; Wang *et al.* 2012) that would have increased their length. Furthermore, the first introns in the CDS and the 5′ untranslated region (which have not been analyzed in this study) of genes have been hypothesized to aid in gene expression (*i.e.*, intron-mediated enhancement), as they contain additional regulatory elements (Bradnam and Korf 2008; Wang *et al.* 2012). This form of expression enhancement has so far only been reported for plants (Parra *et al.* 2011), and further analysis of the sequences of the first introns in the core genes of *Fusarium* species could therefore lead to interesting and insightful information regarding the expression regulation repertoire of these fungi.

In this study, we detected a 5′ positional bias for introns in the majority of *Fusarium* core genes. Lin and Zhang (2005) attributed this to a preferential loss of introns in the 3′ region of genes, especially core genes, during evolution. To explain how such losses may occur, they proposed a mechanism based on homologous recombination between the genomic copies of the genes and their reverse-transcribed spliced mRNAs (Fink 1987), although the molecular basis of this hypothesis remains to be determined. Within the *Fusarium* core gene dataset, there were also some exceptions to the observed 5′ positional bias, especially in genes harboring two and four introns. These genes generally have a near-equal distribution of introns in both the 5′ and the 3′ regions represented in category 3 of Figure 4B, where 50% of the introns are in the 5′ region and 50% are in the 3′ region. A similar trend was previously observed in certain genes of *N. crassa*, *Magnaporthe grisea*, and *F. graminearum*, where it was suggested that intron loss occurred in the middle of the genes (Niu *et al.* 2005).

Our comparison of intron positions in the four *Fusarium* species revealed that the positional conservation extended to the exact phase of the intron. Of the 2022 introns examined, the phase of only two was not conserved between the four *Fusarium* species. But among these introns, phase was nonuniformly distributed with phase 0 introns occurring more often than phase 1 and 2 introns, and phase 1 introns occurring more often than phase 2 introns. Both these findings are consistent with what has been observed before (Irimia and Roy 2008; Fedorov *et al.* 1992; Qiu *et al.* 2004). In addition, most of the phase 0 introns examined for *Fusarium* appeared to be closer to the 5′ end of the genes. The observation of an excess of phase 0 introns in a genome has been used as support for the so-called “introns early” theory, which speculates that introns existed in the progenote before the diversification of the three Domains of Life (Nguyen *et al.* 2006; Roy 2003). Also, 35% of extant introns are phase 0, which is speculated to be an inherited state (Roy 2003; de Souza *et al.* 1998). Although this theory would explain why phase 0 introns are more prevalent than phase 1 and 2 introns, it does not explain why phase 1 introns are more prevalent than phase 2 introns. Therefore, proponents of the “introns late” theory have proposed a site (the “proto-splice site”) in which introns are inserted nonrandomly to explain the high proportion of phase 0 introns, and why there are more phase 1 introns than phase 2 introns (Logsdon 1998; Dibb and Newman 1989; Long *et al.* 1998).

### Refined consensus sequences for cis-elements involved in Fusarium intron splicing

As expected, the 5′ splice site of the *Fusarium* introns was more degenerate than the 3′ splice site and the branching site (Iwata and Gotoh 2011). This is probably due to more nucleotides being part of the motif. However, when comparisons were made between the *Fusarium cis*-elements and those found in the genomes of other fungal genera, differences were found mainly in the 5′ splice site. *Aspergillus fumigatus*, *Candida albicans*, *Cryptococcus neoformans*, *Schizosaccharomyces* *pombe*, and *S*. *cerevisiae* have been reported to have the 5′ splice site sequence A_35_A_39_G_47_|G_100_T_99_R_90_A_56_G_90_T_72_ (Bhasi *et al.* 2007). In a much earlier study, *N. crassa* introns have been reported to have the 5′ splice site sequence G_51_|G_99_T_99_(A_77_/G_17_)(A_50_/C_23_) G_94_ (T_76_/C_15_) (Bruchez *et al.* 1993). These consensus sequences share similarities with what was found in the current study for the four *Fusarium* species (*e.g.*, the nucleotide at the third position within the intron is most frequently a purine). However, as with the study in *N. crassa*, our study provided more information on this consensus sequence (see Figure 5 and Table 1). For example, there was no information on the proportion of each purine at the third position within introns in *As. fumigatus*, *Ca. albicans*, *Cr. neoformans*, *Sc. pombe*, and *S*. *cerevisiae*, and when we decoded this position in our dataset it was observed that a 2′-deoxyadenosine occurs 74% of the time and a 2′-deoxyguanosine 16% of time.

Of the four motifs analyzed in this study, the 3′ splice site was the most conserved. The *Fusarium* 3′ splice site consensus motif (Y_93_A_100_G_100_|R_59_, where subscripts denote frequency) was highly similar to that found in *As. fumigatus*, *Ca. albicans*, *Cr. neoformans*, *Sc. pombe*, and *S. cerevisiae*, with their 3′ splice site consensus motif being Y_89_A_100_G_100_|R_58_ (Kupfer *et al.* 2004; Bhasi *et al.* 2007). These two motifs differ from that of *N. crassa* (Y_92_A_100_G_100_|Y_88_) in the first nucleotide of the 3′ exon, where pyrimidines are more frequent than purines (Bruchez *et al.* 1993). The YAG motif has also been reported for the introns of animals (Mount 1982; Gates *et al.* 2011). In the current study, we observed two additional RAG-containing motifs supported by EST evidence. These were RAG|Y and RAG|R, and they were found in 5.69% of introns examined. Thus, the minimal requirement for a 3′ splice site for all fungal species analyzed thus far is four nucleotides containing a 2′-deoxyadenosine followed by a 2′-deoxyguanosine at the second and third nucleotide positions, respectively.

While the intron branch site motifs examined for the four *Fusarium* species were relatively conserved, some variation was observed. In addition to the most common CTRAY motif, the core gene dataset used in this study also included introns with branch site motifs TTRAY (4.99% of introns) and CTHAH (3.96% of introns), as well as an ACCAT motif that occurred in 0.05% introns. Of these, the TTRAY motif has been reported for other fungi. Kupfer *et al.* (2004) indicated that this sequence represents a secondary fungal branch site motif based on the genomes of *S. cerevisiae*, *Sc. pombe*, *As. nidulans*, *N. crassa*, and *Cr. neoformans*, and it has also been observed in 1 out of 72 genes examined in *N. crassa* (Bruchez *et al.* 1993). To the best of our knowledge, the motifs CTHAH and ACCAT have not yet been reported and whether these motifs are unique to *Fusarium* remains to be confirmed.

The sequences of the 5′ splice site and the branch site define the type of spliceosome needed for the splicing of the introns bearing these *cis*-elements (Wahl *et al.* 2009). During splicing, the U1 and U2 spliceosomal components bind to the 5′ splice site and the branch site in a sequential manner (Russell 2006; Newman and Nagai 2010). In yeast, where the 5′ splice site and the branch site are highly conserved, the sequence of these *cis*-elements is the only defining factor. However, in higher eukaryotes, where these sequences are more degenerate, additional factors such as splicing enhancers and silencers present on the pre-mRNA also influence the type of spliceosome needed (Wahl *et al.* 2009). Since the 5′ splice site has been found to be degenerate and secondary branch site motifs have been found in the four *Fusarium* species examined, pre-mRNA splicing enhancers and silencers could also be involved in the definition of the type of spliceosome needed for splicing. Further research on pre-mRNA splicing enhancers and silencers in *Fusarium* species could shed light on this subject.

Consistent with what has been observed for other fungi (Kupfer *et al.* 2004), the polypyrimidine tract was the most diverse intron *cis*-element examined in the four *Fusarium* species. In the *Fusarium* core gene dataset, this diversity was further emphasized by the multiplicity of potential polypyrimidine tracts predicted for single genes. For example, in one alignment of the core genes of the four species, *F. verticillioides* had six different predicted polypyrimidine tracts, *F. circinatum* nine, *F. oxysporum* four, and *F. graminearum* six. However, the predicted polypyrimidine tracts were predominantly found at the 5′ region of introns in *Fusarium*. This is in contrast to what has been found in animals where the polypyrimidine tract is situated mainly in the 3′ region of introns (Banerjee *et al.* 2004). The predominance of the polypyrimidine tract at the 5′ region of introns has also been reported for the introns of *S. cerevisiae*, *Sc. pombe*, *As. nidulans*, *N. crassa*, and *Cr. neoformans* (Kupfer *et al.* 2004). Such diversity in the sequence, length, and position of the polypyrimidine tract suggests that the spliceosomal machinery for different organisms differ markedly, either in terms of the constituents of the spliceosome itself or in terms of their specificity. For example, experimental work has shown that polypyrimidine tracts are not always essential for splicing, but when they are present, protein U2AF^65^ of the spliceosome binds to it, subsequently allowing more efficient splicing (Banerjee *et al.* 2004). A more detailed analysis of the polypyrimidine tracts in the spliceosomal introns of *Fusarium* and other fungi would undoubtedly shed light not only on the splicing mechanisms in these organisms, but potentially allow the identification of novel regulatory targets for gene expression.

### Conclusions

In this study, we showed that publicly available genome annotations for *F. circinatum*, *F. verticillioides*, *F. oxysporum*, and *F. graminearum* include numerous erroneously annotated introns. Although an annotated reference genome can aid in the annotation of the newly sequenced genome of one or more close relatives (Irimia and Roy 2008), gene prediction software may still make mistakes. This is because the genomes of the relatives may not experience similar evolutionary rates and pressures. Moreover, gene prediction methods used to annotate these sequenced genomes are often developed and trained on an organism that is distantly related to them. For instance, the initial annotations of three *Fusarium* genomes (*F. verticillioides*, *F. oxysporum*, and *F. graminearum*) were done using GENEid (Guigo *et al.* 1992) and FGENESH (Solovyev *et al.* 1994), which were trained on human and vertebrate genes. Also, although these gene prediction methods use algorithms that recognize start and stop codons, the coding region, the acceptor and donor regions, and the 5′ and 3′ intron regions, they do not have robust algorithms for recognizing features such as the branch site of introns.

When the overall intron architecture of the core genes of the four *Fusarium* species was compared, *F. graminearum* often had a different structure. It contributed to ∼60% of the intron position incongruences between the four species and its intron lengths were mostly different to those of the other three species, which is consistent with what has been observed by Croll and McDonald (2012). Also, in instances where the other three species had an alternative branch site sequence, *F. graminearum* had the canonical CTRAY motif, and vice versa. In contrast, the structure of introns in *F. circinatum*, *F. verticillioides*, and *F. oxysporum* often resembled one another, with the structure and sequence of introns in *F. circinatum* being highly similar to those of *F. verticillioides*. Indeed, these similarities and differences reflect the known phylogeny of these fungi (Wingfield *et al.* 2012; Bruchez *et al.* 1993; Kumar *et al.* 2010), which further emphasizes the potential impact of evolutionary relationships when utilizing external evidence for genome annotations.

Incorporation of the findings presented here into gene finding procedures would undoubtedly increase the accuracy of annotations for *Fusarium* species. This is specifically true in terms of the branch site motif (*i.e.*, CTRAY, TTRAY, CTHAH, and ACCAT), the 3′ splice site that should contain a minimum of four nucleotides with the canonical AG dinucleotide at the second and third positions, and the 5′ splice site that should contain a minimum of nine nucleotides: three from the upstream exon, the intron dinucleotide, which can either be a GT or a GC, together with the four nucleotides following it. Our data on the minimal requirements for splicing of the introns could also be used to restrict intron length in gene prediction programs (Lim and Burge 2001; Burge and Karlin 1997; Salamov and Solovyev 2000; Borodovsky and McIninch 1993). Consistent with our findings, Nguyen *et al.* (2006) also suggested that the incorporation of intron-phase prediction algorithms in annotation software can markedly increase accuracy and even help correct sequencing errors that appear as indels or substitutions. Many research projects depend on publicly available genomes, and these improvements to fungal gene prediction methods will reduce the discrepancies resulting from a lack of specificity during fungal genome annotations, thereby increasing their reliability.

## Supplementary Material

Supplemental material is available online at www.g3journal.org/lookup/suppl/doi:10.1534/g3.117.300344/-/DC1.
