# Supplementary material for: Architecture and Distribution of Introns in Core Genes of Four Fusarium Species
Source: G3 (Bethesda). 2017 Oct 9;7(11):3809–20. doi: 10.1534/g3.117.300344 (PMC5677156; doi:10.1534/g3.117.300344)
Supplement: Supplementary file 2 [file 3809FileS2.docx]

**Table S1.** The number of Spliceosomal introns per core gene of *F. verticillioides*, *F. circinatum*, *F. oxysporum* and *F. graminearum*.

| **Gene name^a^** | ***S. cerevisiae* Id no.^b^** | **Biological process^c^** | **Number of introns^d^** |
| --- | --- | --- | --- |
| Pre-mRNA-splicing factor clf1 | YLR117c_KOG1915 | Member of the NineTeen Complex (NTC) that contains Prp19p and stabilizes U6 snRNA in catalytic forms of the spliceosome containing U2, U5, and U6 snRNAs; interacts with U1 snRNP proteins | 0 |
| Aminomethyltransferase mitochondrial precursor | YDR019c_KOG2770 | T subunit of the mitochondrial glycine decarboxylase complex, required for the catabolism of glycine to 5,10-methylene-THF; expression is regulated by levels of levels of 5,10-methylene-THF in the cytoplasm | 0 |
| Seryl-tRNA synthetase | YHR011w_KOG2509 | Probable mitochondrial seryl-tRNA synthetase, mutant displays increased invasive and pseudohyphal growth | 0 |
| Uridylate kinase | YKL024c_KOG3079 | Uridylate kinase, catalyzes the seventh enzymatic step in the de novo biosynthesis of pyrimidines, converting uridine monophosphate (UMP) into uridine-5'-diphosphate (UDP) | 0 |
| DNA repair helicase RAD25 | YIL143c_KOG1123 | Required, with Rad3p, for unwinding promoter DNA; involved in DNA repair | 0 |
| pre mRNA splicing factor Prp31 | YGR091w_KOG2574 | Splicing factor, component of the U4/U6-U5 snRNP complex | 1 |
| Multifunctional methyltransferase subunit  TRM112 | YNR046w_KOG1088 | In combination with Trm9p and Trm11p; subunit of complex with Mtq2p that methylates Sup45p (eRF1) in the ternary complex eRF1-eRF3-GTP; deletion confers resistance to zymocin | 1 |
| 2 oxoglutarate dehydrogenase E1 component mitochondrial precursor | YIL125w_KOG0450 | Component of the mitochondrial alpha-ketoglutarate complex, which catalyzes a key step in the tricarboxylic acid (TCA) cycle, the oxidative decarboxylation of alpha-ketoglutarate to form succinyl-CoA | 1 |
| Geranylgeranyl transferase type-2 subunit beta | YPR176c_KOG0366 | Required for vesicular transport between the endoplasmic reticulum and the Golgi; provides a membrane attachment moiety to Rab-like proteins Ypt1p and Sec4p | 1 |
| Exonuclease | YKL113c_KOG2519 | 5' to 3' exonuclease, 5' flap endonuclease, required for Okazaki fragment processing and maturation as well as for long-patch base-excision repair; member of the S. pombe RAD2/FEN1 family | 1 |
| Methyltransferase | YCR047c_KOG1541 | Methyltransferase, methylates residue G1575 of 18S rRNA; required for rRNA processing and nuclear export of 40S ribosomal subunits independently of methylation activity; diploid mutant displays random budding pattern | 1  [1] |
| Glycerol 3 phosphate dehydrogenase | YOL059w_KOG2711 | NAD-dependent glycerol 3-phosphate dehydrogenase, homolog of Gpd1p, expression is controlled by an oxygen-independent signaling pathway required to regulate metabolism under anoxic conditions; located in cytosol and mitochondria | 1 |
| Superoxide dismutase mitochondrial precursor | YHR008c_KOG0876 | Mitochondrial manganese superoxide dismutase, protects cells against oxygen toxicity | 1 |
| Mitochondrial import inner membrane translocase subunit TIM16 | YJL104w_KOG3442 | Constituent of the import motor (PAM complex)component of the Translocase of the Inner Mitochondrial membrane (TIM23 complex); forms a 1:1 subcomplex with Pam18p and inhibits its cochaperone activity; contains a J-like domain | 1 |
| Mitochondrial inner membrane i-AAA protease supercomplex subunit YME1 | YPR024w_KOG0734 | Essential for degradation of unassembled subunit 2 of cytochrome c oxidase in yeast mitochondria | 1 |
| proteasome component C5 | YBL041w_KOG0179 | Required for the receptor-mediated retrieval of luminal ER proteins from the secretory pathway | 1 |
| 60S acidic ribosomal protein P2-beta | YDR382w_KOG3449 | Ribosomal protein P2 beta, a component of the ribosomal stalk, which is involved in the interaction between translational elongation factors and the ribosome; regulates the accumulation of P1 (Rpp1Ap and Rpp1Bp) in the cytoplasm | 1 |
| Adenosylhomocysteinase | YER043c_KOG1370 | S-adenosyl-L-homocysteine hydrolase, catabolizes S-adenosyl-L-homocysteine which is formed after donation of the activated methyl group of S-adenosyl-L-methionine (AdoMet) to an acceptor | 1**** |
| Dolichyl pyrophosphate Man9GlcNAc2 alpha-1,3-glucosyltransferase | YOR002w_KOG2575 | Involved in transfer of oligosaccharides from dolichyl pyrophosphate to asparagine residues of proteins during N-linked protein glycosylation | 1 |
| Elongation factor Tu domain 2 | YOR187w_KOG0460 | Comprises both GTPase and guanine nucleotide exchange factor activities, while these activities are found in separate proteins in S. pombe and humans | 1 |
| Pyridoxal phosphate binding |  |  | 1 |
| Ubiquinone biosynthesis monooxgenase Coq6 | YGR255c_KOG3855 | Putative flavin-dependent monooxygenase; involved in ubiquinone (Coenzyme Q) biosynthesis; localizes to the matrix face of the mitochondrial inner membrane in a large complex with other ubiquinone biosynthetic enzymes | 1 |
| Serine/threonine-protein kinase KIN28 | YDL108w_KOG0659 | Subunit of the transcription factor TFIIH; involved in transcription initiation at RNA polymerase II promoters | 1 |
| Mannose-6-phosphate isomerase | YER003c_KOG2757 | Catalyzes the interconversion of fructose-6-P and mannose-6-P; required for early steps in protein mannosylation | 1 |
| Mitochondrial-processing peptidase alpha subunit | YHR024c_KOG2067 | Essential processing enzyme that cleaves the N-terminal targeting sequences from mitochondrially imported proteins | 1 |
| U3 small nucleolar RNA-associated protein 7 | YER082c_KOG1272 | Nucleolar protein, component of the small subunit (SSU) processome containing the U3 snoRNA that is involved in processing of pre-18S rRNA | 1 |
| N-acetylglucosamine phosphate mutase | YEL058w_KOG2537 | Essential N-acetylglucosamine-phosphate mutase; converts GlcNAc-6-P to GlcNAc-1-P, which is a precursor for the biosynthesis of chitin and for the formation of N-glycosylated mannoproteins and glycosylphosphatidylinositol anchors | 1 |
| Mitochondrial ornithine transporter 1 | YOR130c_KOG0758 | Ornithine transporter of the mitochondrial inner membrane, exports ornithine from mitochondria as part of  arginine biosynthesis | 1 |
| Galactokinase | YBR020w_KOG0631 | A divergent promoter region in the yeast gal gene cluster is responsible for the LeLoir pathway enzymes necessary for the utilization of galactose | 1 |
| ATP-dependent molecular chaperone HSC82/ Heat shock protein Hsp90 constitutive isoform | YMR186w_KOG0019 | Required in higher concentrations for growth of cells at higher temperatures | 1 |
| Splicing factor 3a subunit 3/ Pre-mRNA-splicing factor PRP9 | YDL030w_KOG2636 | Subunit of the SF3a splicing factor complex,required for spliceosome assembly; acts after the formation of the U1 snRNP-pre-mRNA complex | 1 |
| U3 small nucleolar ribonucleoprotein protein IMP3 | YHR148w_KOG4655 | Component of the SSU processome, which is required for pre-18S rRNA processing, essential protein that interacts with Mpp10p and mediates interactions of Imp4p and Mpp10p with U3 snoRNA | 1 |
| Proteasome component PUP3 | YER094c_KOG0180 | Nucleotide sequence and transcriptional regulation of the yeast recombinational repair gene RAD51 | 1 |
| U3 small nucleolar RNA-associated protein 11 | YKL099c_KOG3237 | Subunit of U3-containing Small Subunit (SSU) processome complex involved in production of 18S rRNA and assembly of small ribosomal subunit | 1 |
| 26S protease regulatory subunit 8 homolog | YGL048c_KOG0728 | Gene encodes a protein homologous to the human Tat-binding protein TBP-1 | 1 |
| Adenine phosphoribosyltransferase | YML022w_KOG1712 | Adenine phosphoribosyltransferase, catalyzes the formation of AMP from adenine and 5-phospho-ribosylpyrophosphate; involved in the salvage pathway of purine nucleotide biosynthesis | 1 |
| Signal recognition particle subunit SRP54 / Signal recognition particle 54 kDa protein homolog | YPR088c_KOG0780 | Essential for growth | 1 |
| Ribosome assembly protein RRB1 | YMR131c_KOG0302 | Essential nuclear protein involved in early steps of ribosome biogenesis; physically interacts with the ribosomal protein Rpl3p | 1 |
| Mitochondrial pyruvate dehydrogenase kinase | YIL042c_KOG0787 | Involved in negative regulation of pyruvate dehydrogenase complex activity by phosphorylating the ser-133 residue of the Pda1p subunit; acts in concert with kinase Pkp2p and phosphatases Ptc5p and Ptc6p | 1 |
| Proteosome component PRE2 precursor | YPR103w_KOG0175 | Beta 5 subunit of the 20S proteasome, responsible for the chymotryptic activity of the proteasome | 1 |
| Pyruvate dehydrogenase E1 component subunit beta | YBR221c_KOG0524 | E1 beta subunit of the pyruvate dehydrogenase (PDH) complex, which is an evolutionarily-conserved multi-protein complex found in mitochondria | 1 |
| ATP dependent protease La/ Lon protease homolog, mitochondrial | YBL022c_KOG2004 | ATP-dependent Lon protease, involved in degradation of misfolded proteins in mitochondria; required for biogenesis and maintenance of mitochondria | 1 |
| GPI anchor transamide precursor | YDR331w_KOG1349 | ER membrane glycoprotein subunit of the glycosyl-phosphatidylinositol transamidase complex that adds glycosylphosphatidylinositol (GPI) anchors to newly synthesized proteins | 1 |
| Ubiquitin-like protein SMT3 | YDR510w_KOG1769 | Ubiquitin-like protein of the SUMO family, conjugated to lysine residues of target proteins; regulates chromatid cohesion, chromosome segregation, APC-mediated proteolysis, DNA replication and septin ring dynamics; phosphorylated at Ser2 | 1 |
| 40S ribosomal protein S9 | YBR189w_KOG3301 | Protein component of the small (40S) ribosomal subunit; nearly identical to Rps9Ap and has similarity to E. coli S4 and rat S9 ribosomal proteins | 2 |
| Proteasome regulatory particle subunit Rpn /26S proteasome regulatory subunit RPN7 | YPR108w_KOG0687 | Essential, non-ATPase regulatory subunit of the 26S proteasome, similar to another S. cerevisiae regulatory subunit, Rpn5p | 2 |
| Histidyl-tRNA synthetase, mitochondrial precursor | YPR033c_KOG1936 | Cytoplasmic and mitochondrial histidine tRNA synthetase; encoded by a single nuclear gene that specifies two messages; efficient mitochondrial localization requires both a presequence and an amino-terminal sequence | 2 |
| Fructose-1, 6-bisphosphatase | YLR377c_KOG1458 | Hey regulatory enzyme in the gluconeogenesis pathway, required for glucose metabolism; undergoes either proteasome-mediated or autophagy-mediated degradation depending on growth conditions; interacts with Vid30p | 2 |
| Phosphomannomutase | YFL045c_KOG3189 | Involved in synthesis of GDP-mannose and dolichol-phosphate-mannose; required for folding and glycosylation of secretory proteins in the ER lumen | 2 |
| AP-1 complex subunit mu-1-I | YPL259c_KOG0937 | Mu1-like medium subunit of the clathrin-associated protein complex (AP-1); binds clathrin; involved in clathrin-dependent Golgi protein sorting | 2*** |
| Ubiquitin fusion degradation protein 1 | YGR048w_KOG1816 | Protein that interacts with Cdc48p and Npl4p, involved in recognition of polyubiquitinated proteins and their presentation to the 26S proteasome for degradation; involved in transporting proteins from the ER to the cytosol | 2 |
| Aspartyl tRNA synthetase | YLL018c_KOG0556 | Primarily cytoplasmic; homodimeric enzyme that catalyzes the specific aspartylation of tRNA(Asp); class II aminoacyl tRNA synthetase; binding to its own mRNA may confer autoregulation | 2**** |
| DNA mismatch repair protein Mlh1 | YMR167w_KOG1979 | Protein required for mismatch repair in mitosis and meiosis as well as crossing over during meiosis; forms a complex with Pms1p and Msh2p-Msh3p during mismatch repair | 2 |
| 60S ribosomal protein L28/ L27a | YGL103w_KOG1742 | Has similarity to E. coli L15 and rat L27a ribosomal proteins; may have peptidyl transferase activity; can mutate to cycloheximide resistance | 2 |
| Vacuolar protein sorting-associated protein 28/ VPS28 | YPL065w_KOG3284 | Component of the ESCRT-I complex (Stp22p, Srn2p, Vps28p, and Mvb12p), which is involved in ubiquitin-dependent sorting of proteins into the endosome; conserved C-terminal domain interacts with ESCRT-III subunit Vps20p | 2  [1] |
| Vacuolar protein sorting-associated protein 21 | YOR089c_KOG0092 | Rab family GTPase required for endocytic transport and for sorting of vacuolar hydrolases; localized in endocytic intermediates; detected in mitochondria; geranylgeranylation required for membrane association | 2 |
| Alanyl-tRNA synthetase; Mitochondrial | YOR335c_KOG0188 | Required for protein synthesis; point mutation (cdc64-1 allele) causes cell cycle arrest at G1 | 2 |
| Replication factor C subunit 5/ RFC5 | YBR087w_KOG2035 | Subunit of heteropentameric Replication factor C (RF-C), which is a DNA binding protein and ATPase that acts as a clamp loader of the proliferating cell nuclear antigen (PCNA) processivity factor for DNA polymerases delta and epsilon | 2 |
| Succinate dehydrogenase [ubiquinone] iron-sulfur subunit, mitochondrial | YLL041c_KOG3049 | Iron-sulfur protein subunit of succinate dehydrogenase (Sdh1p, Sdh2p, Sdh3p, Sdh4p), which couples the oxidation of succinate to the transfer of electrons to ubiquinone as part of the TCA cycle and the mitochondrial respiratory chain | 2 |
| 40S ribosomal protein S18-B | YML026c_KOG3311 | Protein component of the small (40S) ribosomal subunit; nearly identical to Rps18Ap and has similarity to E. coli S13 and rat S18 ribosomal proteins | 2 |
| 60S ribosomal protein L24-A | YGL031c_KOG1722 | Ribosomal protein L30 of the large (60S) ribosomal subunit, nearly identical to Rpl24Bp and has similarity to rat L24 ribosomal protein; not essential for translation but may be required | 2 |
| Protein transport protein SEC61 | YLR378c_KOG1373 | Essential subunit of Sec61 complex (Sec61p, Sbh1p, and Sss1p); forms a channel for SRP-dependent protein import and retrograde transport of misfolded proteins out of the ER; with Sec63 complex allows SRP-independent protein import into ER | 2 |
| Protein sco1 | YBR037c_KOG2792 | Copper-binding protein of the mitochondrial inner membrane, required for cytochrome c oxidase activity and respiration; may function to deliver copper to cytochrome c oxidase; has similarity to thioredoxins | 2 |
| 40S ribosomal protein S19-A | YOL121c_KOG3411 | Protein component of the small (40S) ribosomal subunit, required for assembly and maturation of pre-40 S particles; mutations in human RPS19 are associated with Diamond Blackfan anemia; nearly identical to Rps19Bp | 2 |
| Replication factor C subunit 4 | YOL094c_KOG0991 | Subunit of heteropentameric Replication factor C (RF-C), which is a DNA binding protein and ATPase that acts as a clamp loader of the proliferating cell nuclear antigen (PCNA) processivity factor for DNA polymerases delta and epsilon | 2 |
| Transcription initiation factor IIa small chain | YKL058w_KOG3463 | TFIIA small subunit; involved in transcriptional activation, acts as antirepressor or as coactivator | 2 |
| 60S ribosomal protein L10 | YLR075w_KOG0857 | Protein component of the large (60S) ribosomal subunit, responsible for joining the 40S and 60S subunits; regulates translation initiation; has similarity to rat L10 ribosomal protein and to members of the QM gene family | 2 |
| Phosphoglycerate kinase | YCR012w_KOG1367 | Catalyzes transfer of high-energy phosphoryl groups from the acyl phosphate of 1,3-bisphosphoglycerate to ADP to produce ATP; key enzyme in glycolysis and gluconeogenesis | 2 |
| 26S proteasome non ATPase regulatory subunit 11 | YFR004W | Metalloprotease subunit of the 19S regulatory particle of the 26S proteasome lid; couples the deubiquitination and degradation of proteasome substrates; involved, independent of catalytic activity, in fission of mitochondria and peroxisomes | 2 |
| F actin capping protein subunit beta | YIL034c_KOG3174 | Beta subunit of the capping protein (CP) heterodimer (Cap1p and Cap2p) which binds to the barbed ends of actin filaments preventing further polymerization; localized predominantly to cortical actin patches | 2 |
| Mitochondrial phosphate carrier protein 2 | YER053c_KOG0767 | Imports inorganic phosphate into mitochondria; functionally redundant with Mir1p but less abundant than Mir1p under normal conditions; expression is induced at high temperature | 2 |
| Probable cation-transporting ATPase 1 | YEL031w_KOG0209 | Ion transporter of the ER membrane involved in ER function and Ca2+ homeostasis; required for regulating Hmg2p degradation; confers sensitivity to a killer toxin (SMKT) produced by *Pichia farinosa* KK1 | 2 |
| Small nuclear ribonucleoprotein LSM1  Sm-like protein LSm1? | YJL124c_KOG1782 | Lsm (Like Sm) protein; forms heteroheptameric complex (with Lsm2p, Lsm3p, Lsm4p, Lsm5p, Lsm6p, and Lsm7p) involved in degradation of cytoplasmic mRNAs | 2 |
| ATP-dependent RNA helicase FAL1 | YDR021w_KOG0328 | Nucleolar protein required for maturation of 18S rRNA, member of the eIF4A subfamily of DEAD-box ATP-dependent RNA helicases | 2 |
| Malate dehydrogenase, mitochondrial | YKL085w_KOG1494 | Catalyzes interconversion of malate and oxaloacetate; involved in the tricarboxylic acid (TCA) cycle; phosphorylated | 2 |
| Ribosomal protein S28e/ 40S ribosomal protein S28-A | YOR167c_KOG3502 | Protein component of the small (40S) ribosomal subunit; nearly identical to Rps28Bp | 2 |
| S-adenosylmethionine synthetase | YLR180w_KOG1506 | S-adenosylmethionine synthetase, catalyzes transfer of the adenosyl group of ATP to the sulfur atom of methionine; one of two differentially regulated isozymes (Sam1p and Sam2p) | 2 |
| Eukaryotic translation initiation factor 2 subunit gamma | YER025w_KOG0466 | Involved in the identification of the start codon; binds GTP when forming the ternary complex with GTP and tRNAi-Met | 2  [1] |
| Eukaryotic translation initiation factor 3 subunit G | YDR429c_KOG0122 | eIF3g subunit of the core complex of translation initiation factor 3 (eIF3), which is essential for translation; stimulates resumption of ribosomal scanning during translation reinitiation | 2 |
| Peroxiredoxin TSA1 | YML028w_KOG0852 | Thioredoxin peroxidase, acts as both a ribosome-associated and free cytoplasmic antioxidant; self-associates to form a high-molecular weight chaperone complex under oxidative stress; deletion results in mutator phenotype | 2 |
| Protein farnesyltransferase/geranylgeranyltransferase type-1 subunit alpha | YKL019w_KOG0530 | Catalyzes prenylation of proteins containing a CAAX consensus motif; essential protein required for membrane localization of Ras proteins and a-factor | 2 |
| Protein disulfide-isomerase | YCL043c_KOG0190 | Multifunctional protein resident in the endoplasmic reticulum lumen, essential for the formation of disulfide bonds in secretory and cell-surface proteins, unscrambles non-native disulfide bonds | 2 |
| Proteasome component Pre4 | YFR050c_KOG0185 | Beta 7 subunit of the 20S proteasome | 2 |
| 60S acidic ribosomal protein P0 | YLR340w_KOG0815 | Conserved ribosomal protein P0 of the ribosomal stalk, which is involved in interaction between translational elongation factors and the ribosome | 2 |
| 40S ribosomal protein S7 | YOR096w_KOG3320 | Protein component of the small (40S) ribosomal subunit, nearly identical to Rps7Bp; interacts with Kti11p; deletion causes hypersensitivity to zymocin | 2 |
| rRNA processing protein Rrp20/ Pre-rRNA-processing protein PNO1 | YOR145c_KOG3273 | Essential nucleolar protein required for pre-18S rRNA processing, interacts with Dim1p, an 18S rRNA dimethyltransferase, and also with Nob1p, which is involved in proteasome biogenesis; contains a KH domain | 2 |
| Isocitrate dehydrogenase NADP dependent 1/ Isocitrate dehydrogenase [NADP] cytoplasmic | YLR174w_KOG1526 | Cytosolic NADP-specific isocitrate dehydrogenase, catalyzes oxidation of isocitrate to alpha-ketoglutarate; levels are elevated during growth on non-fermentable carbon sources and reduced during growth on glucose | 2 |
| Heat shock protein SSC1, mitochondrial | YJR045c_KOG0102 | Hsp70 family ATPase, constituent of the import motor component of the Translocase of the Inner Mitochondrial membrane (TIM23 complex); involved in protein translocation and folding; subunit of SceI endonuclease | 2**** |
| 60S ribosomal protein L11-B | YGR085c_KOG0397 | Nearly identical to Rpl11Ap; involved in ribosomal assembly; depletion causes degradation of proteins and RNA of the 60S subunit | 2 |
| Isocitrate dehydrogenase [NAD] subunit 1, mitochondrial | YNL037c_KOG0784 | Catalyzes the oxidation of isocitrate to alpha-ketoglutarate in the TCA cycle | 2 |
| 60S ribosomal protein L15-B | YMR121c_KOG1678 | Protein component of the large (60S) ribosomal subunit, nearly identical to Rpl15Ap | 2 |
| Proteasome component c1 | YOR362c_KOG0184 | Alpha 7 subunit of the 20S proteasome | 2 |
| Peptidyl-prolyl cis-trans isomerase B (cyclophilin) | YHR057c_KOG0880 | Catalyzes the cis-trans isomerization of peptide bonds N-terminal to proline residues; has a potential role in the secretory pathway | 3 |
| Methionine aminopeptidase 1 | YLR244c_KOG2738 | Catalyzes the cotranslational removal of N-terminal methionine from nascent polypeptides; function is partially redundant with that of Map2p | 3 |
| T complex protein 1 subunit eta | YJL111w_KOG0361 | Subunit of the cytosolic chaperonin Cct ring complex, related to Tcp1p, required for the assembly of actin and tubulins in vivo; mutant has increased aneuploidy tolerance | 3 |
| 60S ribosomal protein L37-A | YLR185w_KOG3475 | Has similarity to Rpl37Bp and to rat L37 ribosomal protein | 3 |
| Serine palmitoyltransferase 1 | YMR296c_KOG1358 | Component of serine palmitoyltransferase, responsible along with Lcb2p for the first committed step in sphingolipid synthesis, which is the condensation of serine with palmitoyl-CoA to form 3-ketosphinganine | 3 |
| Structural maintenance of chromosomes protein 3 | YJL074c_KOG0964 | Subunit of the multiprotein cohesin complex required for sister chromatid cohesion in mitotic cells; also required, with Rec8p, for cohesion and recombination during meiosis; phylogenetically conserved SMC chromosomal ATPase family member | 3 |
| 40S ribosomal protein S13 | YDR064w_KOG0400 | Protein component of the small (40S) ribosomal subunit; has similarity to E. coli S15 and rat S13 ribosomal proteins | 3****(4) |
| Methionine aminopeptidase 2 | YBL091c_KOG2775 | Protein involved in regulation of phospholipid metabolism; homolog of Scs2p | 3 |
| Mannose-1-phosphate guanyltransferase (GDP-mannose pyrophosphorylase) | YDL055c_KOG1322 | Synthesizes GDP-mannose from GTP and mannose-1-phosphate in cell wall biosynthesis; required for normal cell wall structure | 3 |
| Triosephosphate isomerase | YDR050c_KOG1643 | Abundant glycolytic enzyme; mRNA half-life is regulated by iron availability; transcription is controlled by activators Reb1p, Gcr1p, and Rap1p through binding sites in the 5' non-coding region | 3 |
| Trafficking protein particle complex subunit BET3 | YKR068c_KOG3330 | Hydrophilic protein that acts in conjunction with SNARE proteins in targeting and fusion of ER to Golgi transport vesicles; component of the TRAPP (transport protein particle) complex | 3 |
| Clathrin heavy chain | YGL206c_KOG0985 | Subunit of the major coat protein involved in intracellular protein transport and endocytosis; two heavy chains form the clathrin triskelion structural component; the light chain (CLC1) is thought to regulate function | 3 |
| 26S protease regulatory subunit 6B homolog | YDR394w_KOG0727 | One of six ATPases of the 19S regulatory particle of the 26S proteasome involved in the degradation of ubiquitinated substrates; substrate of N-acetyltransferase B | 3 |
| 40S ribosomal protein S15 | YOL040c_KOG0898 | Has similarity to E. coli S19 and rat S15 ribosomal proteins | 3 |
| AP-2 complex subunit alpha | YBL037w_KOG1077 | Large subunit of the clathrin associated protein complex (AP-2); involved in vesicle mediated transport | 3 |
| ATP synthase subunit 5; mitochondrial | YDR298c_KOG1662 | Subunit 5 of the stator stalk of mitochondrial F1F0 ATP synthase, which is an evolutionarily conserved enzyme complex required for ATP synthesis; phosphorylated | 3 |
| Centromere/microtubule-binding protein CBF5 | YLR175w_KOG2529 | Pseudouridine synthase catalytic subunit of box H/ACA small nucleolar ribonucleoprotein particles (snoRNPs), acts on both large and small rRNAs and on snRNA U2 | 3  [1] |
| Protein translation factor SUI1 / Eukaryotic translation initiation factor eIF-1 | YNL244c_KOG1770 | Translation initiation factor eIF1; component of a complex involved in recognition of the initiator codon; modulates translation accuracy at the initiation phase | 3 |
| V-type proton ATPase subunit c'' | YHR026w_KOG0233 | Functions in acidification of the vacuole; one of three proteolipid subunits of the V0 domain | 3 |
| ER lumen protein retaining receptor | YBL040c_KOG3106 | HDEL receptor, an integral membrane protein that binds to the HDEL motif in proteins destined for retention in the endoplasmic reticulum; has a role in maintenance of normal levels of ER-resident proteins | 3 |
| Serine/threonine protein phosphatase PP1-1 | YDL047w_KOG0373 | Type 2A-related serine-threonine phosphatase that functions in the G1/S transition of the mitotic cycle; cytoplasmic and nuclear protein that modulates functions mediated by Pkc1p including cell wall and actin cytoskeleton organization | 3 |
| Coatomer subunit beta | YDR238c_KOG1058 | Essential beta-coat protein of the COPI coatomer, involved in ER-to-Golgi protein trafficking and maintenance of normal ER morphology; shares 43% sequence identity with mammalian beta-coat protein (beta-COP) | 3 |
| Small nuclear ribonucleoprotein Sm D2 | YLR275w_KOG3459 | Core Sm protein Sm D2; part of heteroheptameric complex (with Smb1p, Smd1p, Smd3p, Sme1p, Smx3p, and Smx2p) that is part of the spliceosomal U1, U2, U4, and U5 snRNPs | 3 |
| Histone deacetylase RPD3 | YNL330c_KOG1342 | Regulates transcription, silencing, and other processes by influencing chromatin remodeling; forms at least two different complexes which have distinct functions and members | 3*** |
| 40S ribosomal protein S14-A | YCR031c_KOG0407 | Ribosomal protein 59 of the small subunit, required for ribosome assembly and 20S pre-rRNA processing; mutations confer cryptopleurine resistance; nearly identical to Rps14Bp and similar to *E. coli* S11 and rat S14 ribosomal protein | 3 |
| Glucosamine--fructose-6-phosphate aminotransferase [isomerizing] | YKL104c_KOG1268 | Catalyzes the formation of glucosamine-6-P and glutamate from fructose-6-P and glutamine in the first step of chitin biosynthesis | 3 |
| Neutral trehalase | YDR001c_KOG0602 | Degrades trehalose; required for thermotolerance and may mediate resistance to other cellular stresses; may be phosphorylated by Cdc28p | 3 |
| Elongation factor 1-beta | YAL003w_KOG1668 | Translation elongation factor 1 beta; stimulates nucleotide exchange to regenerate EF-1 alpha-GTP for the next elongation cycle; part of the EF-1 complex, which facilitates binding of aminoacyl-tRNA to the ribosomal A site | 3 |
| DNA polymerase  subunit | YJR006w_KOG2732 | DNA polymerase III (delta) subunit, essential for cell viability; involved in DNA replication and DNA repair | 3  [1] |
| Cytochrome c1 mitochondrial precursor/ Cytochrome c1, heme protein, mitochondrial | YOR065w_KOG3052 | Component of the mitochondrial respiratory chain; expression is regulated by the heme-activated, glucose-repressed Hap2p/3p/4p/5p CCAAT-binding complex | 3 |
| ATP synthase gamma chain mitochondrial precursor/ ATP synthase subunit gamma, mitochondrial | YBR039w_KOG1531 | Gamma subunit of the F1 sector of mitochondrial F1F0 ATP synthase, which is a large, evolutionarily conserved enzyme complex required for ATP synthesis | 3 |
| pfkB family carbohydrate kinase |  |  | 3 |
| Protein SYL1 | YDR189w_KOG1301 | Hydrophilic protein involved in vesicle trafficking between the ER and Golgi; SM (Sec1/Munc-18) family protein that binds the tSNARE Sed5p and stimulates its assembly into a trans-SNARE membrane-protein complex | 4 |
| 60S ribosomal protein L16-A | YIL133c_KOG3204 | N-terminally acetylated protein component of the large (60S) ribosomal subunit, binds to 5.8 S rRNA; transcriptionally regulated by Rap1p | 4 |
| ATP dependent RNA helicase SUB2 | YDL084w_KOG0329 | Component of the TREX complex required for nuclear mRNA export; member of the DEAD-box RNA helicase superfamily and is involved in early and late steps of spliceosome assembly | 4 |
| Heat shock 70 kDA protein/ Heat shock protein SSC1, mitochondrial/ mtHSP70 | YJR045c_KOG0102 | Hsp70 family ATPase, constituent of the import motor component of the Translocase of the Inner Mitochondrial membrane (TIM23 complex); involved in protein translocation and folding; subunit of SceI endonuclease | 4 |
| 60S ribosomal protein L13-B | YMR142c_KOG3295 | Protein component of the large (60S) ribosomal subunit, nearly identical to Rpl13Ap; not essential for viability; has similarity to rat L13 ribosomal protein | 4 |
| Leukotriene A 4 hydrolase | YNL045w_KOG1047 | Leucyl aminopeptidase yscIV (leukotriene A4 hydrolase) with epoxide hydrolase activity, metalloenzyme containing one zinc atom; green fluorescent protein (GFP)-fusion protein localizes to the cytoplasm and nucleus | 4**** |
| CaaX farnesyltransferase beta subunit | YDL090c_KOG0365 | Prenylates the a-factor mating pheromone and Ras proteins; required for the membrane localization of Ras proteins and a-factor | 4 |
| RNA polymerase III subunit Rpc25 | YKL144c_KOG3297 | RNA polymerase III subunit C25, required for transcription initiation; forms a heterodimer with Rpc17p; paralog of Rpb7p | 4 |
| Cohesin complex subunit Psm1/ Structural maintenance of chromosomes protein 1 | YFL008w_KOG0018 | Subunit of the multiprotein cohesin complex, essential protein involved in chromosome segregation and in double-strand DNA break repair; SMC chromosomal ATPase family member, binds DNA with a preference for DNA with secondary structure | 4 |
| Proteasome subunit alpha type 6/ Proteasome component C7-alpha | YGL011c_KOG0182 | Alpha 1 subunit of the 20S proteasome involved in the degradation of ubiquitinated substrates; 20S proteasome is the core complex of the 26S proteasome; essential for growth; detected in the mitochondria | 4  [1] |
| Coatomer subunit beta | YDR238c_KOG1058 | Essential beta-coat protein of the COPI coatomer, involved in ER-to-Golgi protein trafficking and maintenance of normal ER morphology | 4 |
| ATP synthase delta chain mitochondrial precursor/ ATP synthase subunit 5, mitochondrial | YDR298c_KOG1662 | Subunit 5 of the stator stalk of mitochondrial F1F0 ATP synthase, which is an evolutionarily conserved enzyme complex required for ATP synthesis; phosphorylated | 4 |
| GTP-binding protein YPT1 | YFL038c_KOG0084 | Rab family GTPase, involved in the ER-to-Golgi step of the secretory pathway; complex formation with the Rab escort protein Mrs6p is required for prenylation of Ypt1p by protein geranylgeranyltransferase type II (Bet2p-Bet4p) | 4 |
| Cell cycle control protein Cwf8 (Pre-mRNA-splicing factor 19???) | YLL036c_KOG0289 | Splicing factor associated with the spliceosome; contains a U-box, a motif found in a class of ubiquitin ligases | 4 |
| Oligosaccharyl transferase stt3 subunit | YGL022w_KOG2292 | Catalyzes asparagine-linked glycosylation of newly synthesized proteins; forms a subcomplex with Ost3p and Ost4p and is directly involved in catalysis | 4 |
| Guanine nucleotide-binding protein subunit beta-like protein | YMR116c_KOG0279 | G-protein beta subunit and guanine nucleotide dissociation inhibitor for Gpa2p; ortholog of RACK1 that inhibits translation; core component of the small (40S) ribosomal subunit; represses Gcn4p in the absence of amino acid starvation | 4 |
| Translationally controlled tumor protein homolog | YKL056c_KOG1727 | Protein that associates with ribosomes; green fluorescent protein (GFP)-fusion protein localizes to the cytoplasm and relocates to the mitochondrial outer surface upon oxidative stress | 4 |
| AP-1 complex subunit sigma-1 | YLR170c_KOG0934 | Small subunit of the clathrin-associated adaptor complex AP-1, which is involved in protein sorting at the trans-Golgi network | 4 |
| Isocitrate dehydrogenase [NAD] subunit 1, mitochondrial | YNL037c_KOG0784 | Subunit of mitochondrial NAD(+)-dependent isocitrate dehydrogenase, which catalyzes the oxidation of isocitrate to alpha-ketoglutarate in the TCA cycle | 4 |
| Phosphoglucomutase-2 | YMR105c_KOG0625 | Catalyzes the conversion from glucose-1-phosphate to glucose-6-phosphate, which is a key step in hexose metabolism; functions as the acceptor for a Glc-phosphotransferase | 4  [1] |
| Heat shock protein homolog SSE1 | YPL106c_KOG0103 | ATPase that is a component of the heat shock protein Hsp90 chaperone complex; binds unfolded proteins; member of the heat shock protein 70 (HSP70) family; localized to the cytoplasm | 5 |
| Transaldolase | YLR354c_KOG2772 | Transaldolase, enzyme in the non-oxidative pentose phosphate pathway; converts sedoheptulose 7-phosphate and glyceraldehyde 3-phosphate to erythrose 4-phosphate and fructose 6-phosphate | 5 |
| T complex protein 1 subunit zeta | YDR188w_KOG0359 | Subunit of the cytosolic chaperonin Cct ring complex, related to Tcp1p, essential protein that is required for the assembly of actin and tubulins in vivo; contains an ATP-binding motif | 5  [1] |
| Proteosome component PRE3 | YJL001w_KOG0174 | Beta 1 subunit of the 20S proteasome, responsible for cleavage after acidic residues in peptides | 5 |
| Coatomer subunit alpha | YDL145c_KOG0292 | Alpha subunit of COPI vesicle coatomer complex, which surrounds transport vesicles in the early secretory pathway | 5 |
| Hit family protein 1 | YDL125c_KOG3275 | Adenosine 5'-monophosphoramidase; interacts physically and genetically with Kin28p, a CDK and TFIIK subunit, and genetically with CAK1; member of the histidine triad (HIT) superfamily of nucleotide-binding proteins and similar to Hint | 5 |
| pac10 protein/ Prefoldin subunit 3 | YGR078c_KOG3313 | Part of the heteromeric co-chaperone GimC/prefoldin complex, which promotes efficient protein folding | 5 |
| Mitotic spindle checkpoint component MAD2 | YJL030w_KOG3285 | Delays the onset of anaphase in cells with defects in mitotic spindle assembly; forms a complex with Mad1p | 5 |
| GTP-binding protein YPT6 | YLR262c_KOG0094 | Rab family GTPase, Ras-like GTP binding protein involved in the secretory pathway, required for fusion of endosome-derived vesicles with the late Golgi, maturation of the vacuolar carboxypeptidase Y | 5 |
| T-complex protein 1 subunit gamma | YJL014w_KOG0364 | Subunit of the cytosolic chaperonin Cct ring complex, related to Tcp1p, required for the assembly of actin and tubulins in vivo | 5 |
| Inosine triphosphate pyrophosphatase | YJR069c_KOG3222 | Conserved protein with deoxyribonucleoside triphosphate pyrophosphohydrolase activity, mediates exclusion of noncanonical purines from deoxy-ribonucleoside triphosphate pools; mutant is sensitive to the base analog 6-N-hydroxylaminopurine | 5**** |
| FK506-binding protein 1B | YNL135c_KOG0544 | Peptidyl-prolyl cis-trans isomerase (PPIase), binds to the drugs FK506 and rapamycin; also binds to the nonhistone chromatin binding protein Hmo1p and may regulate its assembly or function | 5**** |
| ATP synthase subunit beta | YJR121w_KOG1350 | Beta subunit of the F1 sector of mitochondrial F1F0 ATP synthase, which is a large, evolutionarily conserved enzyme complex required for ATP synthesis; phosphorylated | 6*** |
| Glucose-6-phosphate 1-dehydrogenase (G6PD) | YNL241c_KOG0563 | Catalyzes the first step of the pentose phosphate pathway; involved in adapting to oxidatve stress | 6****(4) |
| Protein transport protein SEC23 | YPR181c_KOG1986 | GTPase-activating protein, stimulates the GTPase activity of Sar1p; component of the Sec23p-Sec24p heterodimer of the COPII vesicle coat, involved in ER to Golgi transport | 6 |
| Ribose-phosphate pyrophosphokinase 3 | YHL011c_KOG1448 | 5-phospho-ribosyl-1(alpha)-pyrophosphate synthetase, is required for nucleotide, histidine, and tryptophan biosynthesis; one of five related enzymes, which are active as heteromultimeric complexes | 6 |
| ATP synthase subunit alpha, mitochondrial | YBL099w_KOG1353 | Alpha subunit of the F1 sector of mitochondrial F1F0 ATP synthase, which is a large, evolutionarily conserved enzyme complex required for ATP synthesis; phosphorylated | 6**(7),***(7) |
| V-type proton ATPase subunit B | YBR127c_KOG1351 | Subunit B of the eight-subunit V1 peripheral membrane domain of the vacuolar H+-ATPase (V-ATPase), an electrogenic proton pump found throughout the endomembrane system; contains nucleotide binding sites; also detected in the cytoplasm | 6 |
| Protein phosphatase PP2A regulatory subunit A | YAL016w_KOG0211 | Regulatory subunit A of the heterotrimeric protein phosphatase 2A (PP2A), which also contains regulatory subunit Cdc55p and either catalytic subunit Pph21p or Pph22p; required for cell morphogenesis and transcription by RNA polymerase III | 7  [1] |
| Glutamine-dependent NAD (+) synthetase | YHR074w_KOG2303 | Essential for the formation of NAD(+) from nicotinic acid adenine dinucleotide | 14****(12) |
| CTP synthase 2 | YJR103w_KOG2387 | Minor CTP synthase isozyme (see also URA7), catalyzes the ATP-dependent transfer of the amide nitrogen from glutamine to UTP, forming CTP, the final step in de novo biosynthesis of pyrimidines; involved in phospholipid biosynthesis | 15  [2] |

^a^Gene names and ^c^Biological process are described as in the Gene Ontology Databse AmiGo (<http://amigo.geneontology.org/cgi-bin/amigo/gp-details.cgi?gp=SGD:S000006380&session_id=8797amigo1327045088>).

^b^*S. cerevisiae* Id no. indicates the *S. cerevisiae* identity numbers as found in CEGEMA.

^d^Asterisks show genes in which there was a difference in intron number within the four *Fusarium* species where one to four asterisks respectively indicates that the intron had a divergent position in *F. verticillioides*, *F. circinatum*, *F. oxysporum* or *F. graminearum*. The numbers in parentheses indicates the variant intron number for the respective genes and species. The EST data for this positional congruence analysis were obtained for *F. verticillioides*, *F. oxysporum* and *F. graminearum* from the Broad Institute. For *F. circinatum*, EST data were obtained from NCBI (accession number SRR1168456), and an additional unpublished set of EST data for this fungus were obtained from S.L. Slinski and T.R. Gordon (University of California, Davis). The numbers in square brackets indicate the number of introns for the 226 core gene dataset for which positional incongruence (detected during the 2012 study) was eliminated following the February 2014 re-analysis against the Broad Institute.

**Table S3.** The percentage of introns with polypyrimidine tracts (PPTs) in the 5ꞌ region only, in the 3ꞌ region only, and in both the 5ꞌ and 3ꞌ regions.

| **Species** | **% of introns with PPTs in 5ꞌ region** | **% of introns with PPTs in 3ꞌ region** | **% of introns with PPTs in both the 5ꞌ and 3ꞌ regions** |
| --- | --- | --- | --- |
| *F. verticillioides* | 60 | 5 | 35 |
| *F. circinatum* | 58 | 11 | 31 |
| *F. oxysporum* | 70 | 0 | 30 |
| *F.graminearum* | 55 | 0 | 45 |

# The 5′ region is the region between the 5′ splice site and the branch site and the 3′ region is the region between the branch site and the 3′ splice site. PPTs = polypyrimidine tracts.
