# Supplementary material for: Architecture and Distribution of Introns in Core Genes of Four Fusarium Species
Source: G3 (Bethesda). 2017 Oct 9;7(11):3809–20. doi: 10.1534/g3.117.300344 (PMC5677156; doi:10.1534/g3.117.300344)
Supplement: Supplementary file 3 [file 3809FileS3.pptx]

## Slide 1
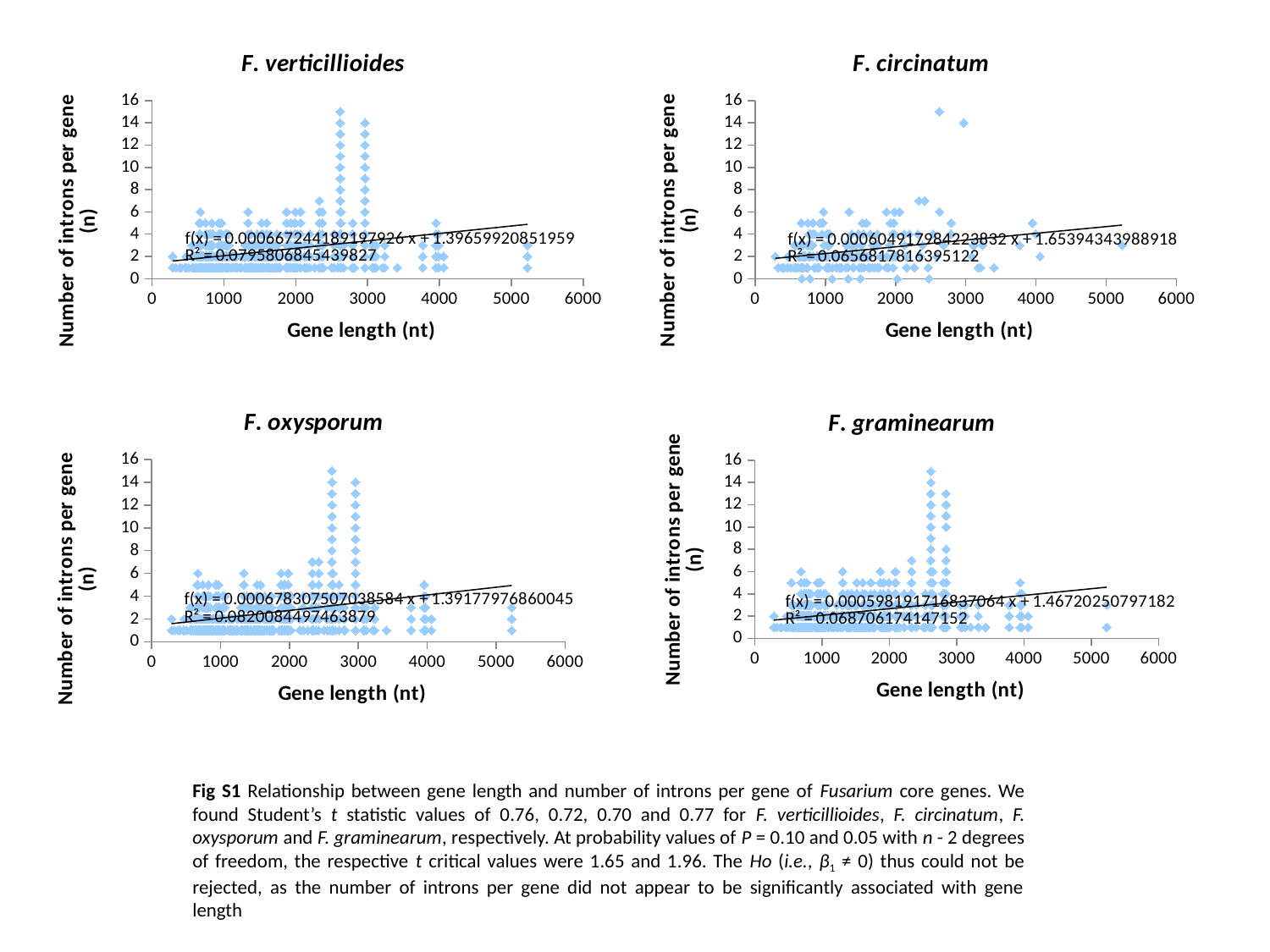

### Chart: F. verticillioides
| Category | Intron no. |
|---|---|
### Chart: F. circinatum
| Category | Number of introns per gene |
|---|---|
### Chart: F. oxysporum
| Category | Intron no. |
|---|---|
### Chart: F. graminearum
| Category | Intron no. |
|---|---|Fig S1 Relationship between gene length and number of introns per gene of Fusarium core genes. We found Student’s t statistic values of 0.76, 0.72, 0.70 and 0.77 for F. verticillioides, F. circinatum, F. oxysporum and F. graminearum, respectively. At probability values of P = 0.10 and 0.05 with n - 2 degrees of freedom, the respective t critical values were 1.65 and 1.96. The Ho (i.e., β1 ≠ 0) thus could not be rejected, as the number of introns per gene did not appear to be significantly associated with gene length

## Slide 2
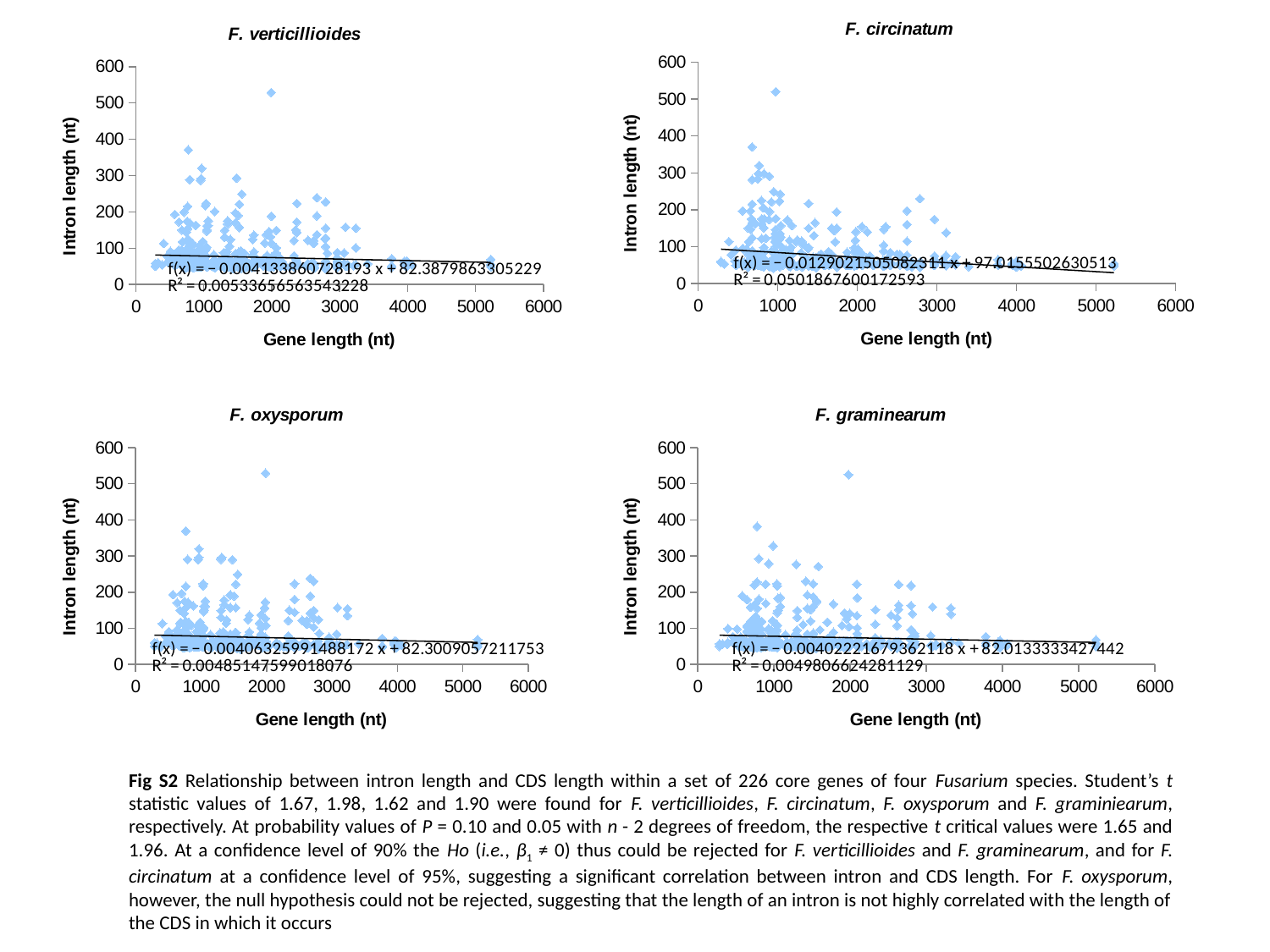

### Chart: F. circinatum
| Category | intron length (nt) |
|---|---|
### Chart: F. verticillioides
| Category | intron length (nt) |
|---|---|
### Chart: F. oxysporum
| Category | intron length (nt) |
|---|---|
### Chart: F. graminearum
| Category | intron length (nt) |
|---|---|Fig S2 Relationship between intron length and CDS length within a set of 226 core genes of four Fusarium species. Student’s t statistic values of 1.67, 1.98, 1.62 and 1.90 were found for F. verticillioides, F. circinatum, F. oxysporum and F. graminiearum, respectively. At probability values of P = 0.10 and 0.05 with n - 2 degrees of freedom, the respective t critical values were 1.65 and 1.96. At a confidence level of 90% the Ho (i.e., β1 ≠ 0) thus could be rejected for F. verticillioides and F. graminearum, and for F. circinatum at a confidence level of 95%, suggesting a significant correlation between intron and CDS length. For F. oxysporum, however, the null hypothesis could not be rejected, suggesting that the length of an intron is not highly correlated with the length of the CDS in which it occurs

## Slide 3
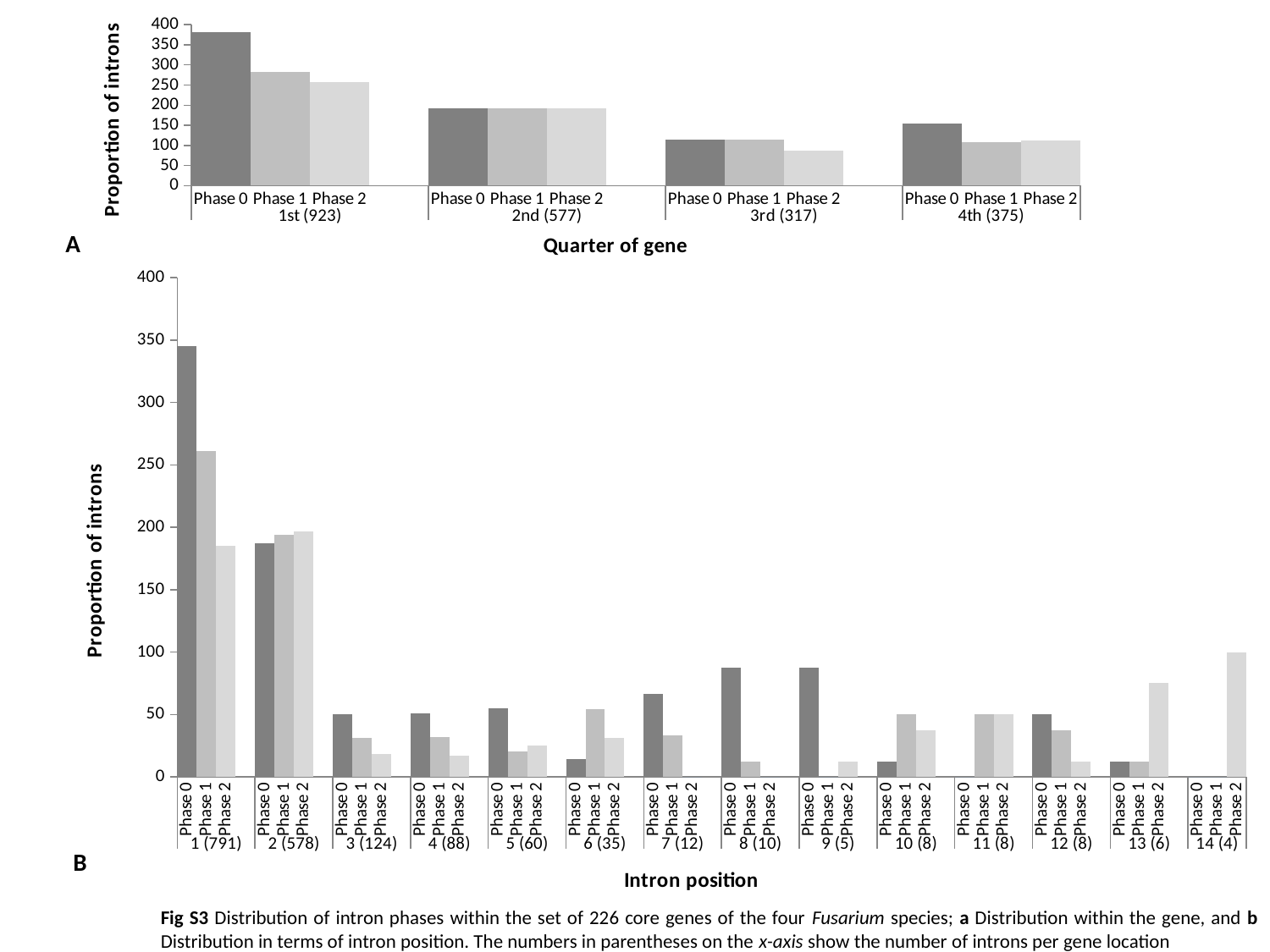

### Chart
| Category | Proportion of introns |
|---|---|
| Phase 0 | 382.0 |
| Phase 1 | 283.0 |
| Phase 2 | 258.0 |
| | None |
| Phase 0 | 193.0 |
| Phase 1 | 192.0 |
| Phase 2 | 192.0 |
| | None |
| Phase 0 | 114.0 |
| Phase 1 | 115.0 |
| Phase 2 | 88.0 |
| | None |
| Phase 0 | 154.0 |
| Phase 1 | 108.0 |
| Phase 2 | 113.0 |A
### Chart
| Category | Proportion of introns |
|---|---|
| Phase 0 | 345.0 |
| Phase 1 | 261.0 |
| Phase 2 | 185.0 |
| | None |
| Phase 0 | 187.0 |
| Phase 1 | 194.0 |
| Phase 2 | 197.0 |
| | None |
| Phase 0 | 50.0 |
| Phase 1 | 31.45 |
| Phase 2 | 18.55 |
| | None |
| Phase 0 | 51.14 |
| Phase 1 | 31.82 |
| Phase 2 | 17.05 |
| | None |
| Phase 0 | 54.82 |
| Phase 1 | 20.13 |
| Phase 2 | 25.05 |
| | None |
| Phase 0 | 14.24 |
| Phase 1 | 54.52 |
| Phase 2 | 31.25 |
| | None |
| Phase 0 | 66.67 |
| Phase 1 | 33.33 |
| Phase 2 | 0.0 |
| | None |
| Phase 0 | 87.5 |
| Phase 1 | 12.5 |
| Phase 2 | 0.0 |
| | None |
| Phase 0 | 87.5 |
| Phase 1 | 0.0 |
| Phase 2 | 12.5 |
| | None |
| Phase 0 | 12.5 |
| Phase 1 | 50.0 |
| Phase 2 | 37.5 |
| | None |
| Phase 0 | 0.0 |
| Phase 1 | 50.0 |
| Phase 2 | 50.0 |
| | None |
| Phase 0 | 50.0 |
| Phase 1 | 37.5 |
| Phase 2 | 12.5 |
| | None |
| Phase 0 | 12.5 |
| Phase 1 | 12.5 |
| Phase 2 | 75.0 |
| | None |
| Phase 0 | 0.0 |
| Phase 1 | 0.0 |
| Phase 2 | 100.0 |Fig S3 Distribution of intron phases within the set of 226 core genes of the four Fusarium species; a Distribution within the gene, and b Distribution in terms of intron position. The numbers in parentheses on the x-axis show the number of introns per gene location

## Slide 4
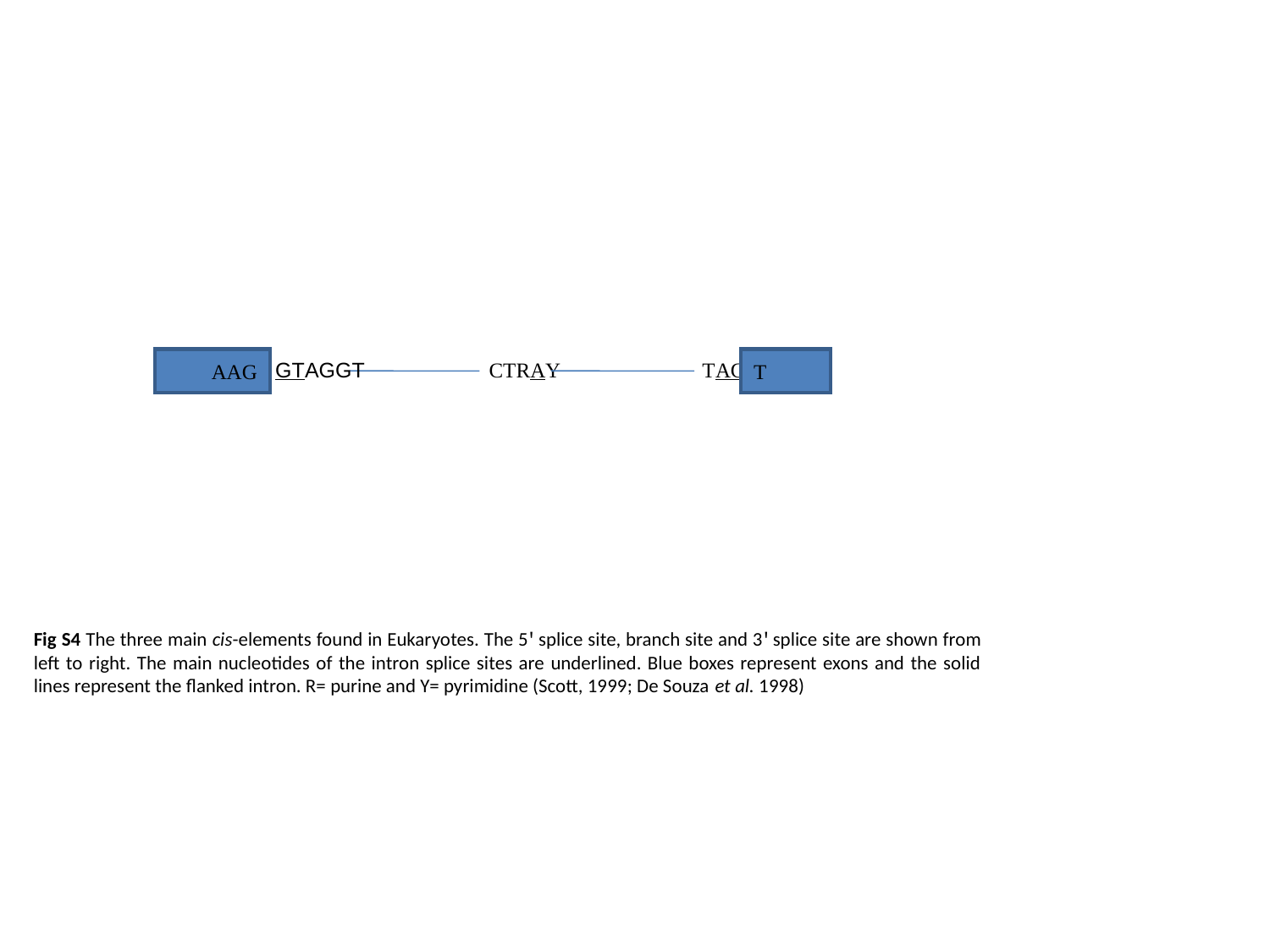

GTAGGT CTRAY TAG
AAG
T
Fig S4 The three main cis-elements found in Eukaryotes. The 5ꞌ splice site, branch site and 3ꞌ splice site are shown from left to right. The main nucleotides of the intron splice sites are underlined. Blue boxes represent exons and the solid lines represent the flanked intron. R= purine and Y= pyrimidine (Scott, 1999; De Souza et al. 1998)
