# Supplementary material for: Architecture and Distribution of Introns in Core Genes of Four Fusarium Species
Source: G3 (Bethesda). 2017 Oct 9;7(11):3809–20. doi: 10.1534/g3.117.300344 (PMC5677156; doi:10.1534/g3.117.300344)
Supplement: Supplementary file 5 [file 3809FileS5.docx]

**Supplementary data**

**File S1** fasta

Alignments of 152 genes whose errors have been resolved with respect to intron position using Augustus

**File S2** docx

**Table S1** The number of spliceosomal introns per core gene of *F. verticillioides*, *F. circinatum*, *F. oxysporum* and *F. graminearum*

**Table S3** The percentage of introns with polypyrimidine tracts (PPTs) in the 5ꞌ region only, in the 3ꞌ region only, and in both the 5ꞌ and 3ꞌ regions

**File S3** pptx

**Fig S1** Relationship between gene length and number of introns per gene of *Fusarium* core genes. We found Student’s *t* statistic values of 0.76, 0.72, 0.70 and 0.77 for *F. verticillioides*, *F. circinatum*, *F. oxysporum* and *F. graminearum*, respectively. At probability values of *P* = 0.10 and 0.05 with *n* - 2 degrees of freedom, the respective *t* critical values were 1.65 and 1.96. The *Ho* (*i.e.*, *β*_1_ ≠ 0) thus could not be rejected, as the number of introns per gene did not appear to be significantly associated with gene length

**Fig S2** Relationship between intron length and CDS length within a set of 226 core genes of four *Fusarium* species. Student’s *t* statistic values of 1.67, 1.98, 1.62 and 1.90 were found for *F. verticillioides*, *F. circinatum*, *F. oxysporum* and *F. graminiearum*, respectively. At probability values of *P* = 0.10 and 0.05 with *n* - 2 degrees of freedom, the respective *t* critical values were 1.65 and 1.96. At a confidence level of 90% the *Ho* (*i.e.*, *β*_1_ ≠ 0) thus could be rejected for *F. verticillioides* and *F. graminearum*, and for *F. circinatum* at a confidence level of 95%, suggesting a significant correlation between intron and CDS length. For *F. oxysporum*, however, the null hypothesis could not be rejected, suggesting that the length of an intron is not highly correlated with the length of the CDS in which it occurs

**Fig S3** Distribution of intron phases within the set of 226 core genes of the four *Fusarium* species; **a** Distribution within the gene, and **b** Distribution in terms of intron position. The numbers in parentheses on the *x-axis* show the number of introns per gene location

**Fig S4** The three main *cis*-elements found in Eukaryotes. The 5ꞌ splice site, branch site and 3ꞌ splice site are shown from left to right. The main nucleotides of the intron splice sites are underlined. Blue boxes represent exons and the solid lines represent the flanked intron. R= purine and Y= pyrimidine (Scott, 1999; De Souza *et al.* 1998)

**File S4** xlsx

**Table S2** Spread sheet with raw data for the intron attributes examined in the set of 226 core genes for the four *Fusarium* species. These data include intron and gene lengths (nt); intron number per gene; intron position in gene; 5ꞌ splice site, polypyrimidine tract, branch site and 3ꞌ splice site sequences; polypyrimidine tract and branch site positions in a gene; and EST data availability for each gene
